# Reliable and robust control of nucleus centering is contingent on nonequilibrium force patterns

**DOI:** 10.1016/j.isci.2023.106665

**Published:** 2023-04-13

**Authors:** Ishutesh Jain, Madan Rao, Phong T. Tran

**Affiliations:** 1 Institut Curie, PSL Universite, Sorbonne Universite, CNRS UMR 144, 75005 Paris, France; 2 Simons Centre for the Study of Living Machines, National Centre for Biological Sciences - TIFR, Bangalore 560065, India; 3 Department of Cell and Developmental Biology, University of Pennsylvania, Philadelphia, PA 19104, USA

**Keywords:** Biological sciences, Microbiology, Molecular biology, Molecular microbiology

## Abstract

Cell centers their division apparatus to ensure symmetric cell division, a challenging task when the governing dynamics is stochastic. Using fission yeast, we show that the patterning of nonequilibrium polymerization forces of microtubule (MT) bundles *controls* the precise localization of spindle pole body (SPB), and hence the division septum, at the onset of mitosis. We define two cellular objectives, *reliability*, the mean SPB position relative to the geometric center, and *robustness*, the variance of the SPB position, which are sensitive to genetic perturbations that change cell length, MT bundle number/orientation, and MT dynamics. We show that simultaneous control of reliability and robustness is required to minimize septum positioning error achieved by the wild type (WT). A stochastic model for the MT-based nucleus centering, with parameters measured directly or estimated using Bayesian inference, recapitulates the maximum fidelity of WT. Using this, we perform a sensitivity analysis of the parameters that control nuclear centering.

## Highlights

• Nucleus positioning is sensitive to the patterns of microtubule polymerization forces
• Perturbations in force pattern affect two objectives: reliability and robustness
• Co-optimization of reliability and robustness reduces error in septum localization
• Numerical simulations and sensitivity analysis reveal positioning control mechanisms

## Graphical abstract

**Figure:**
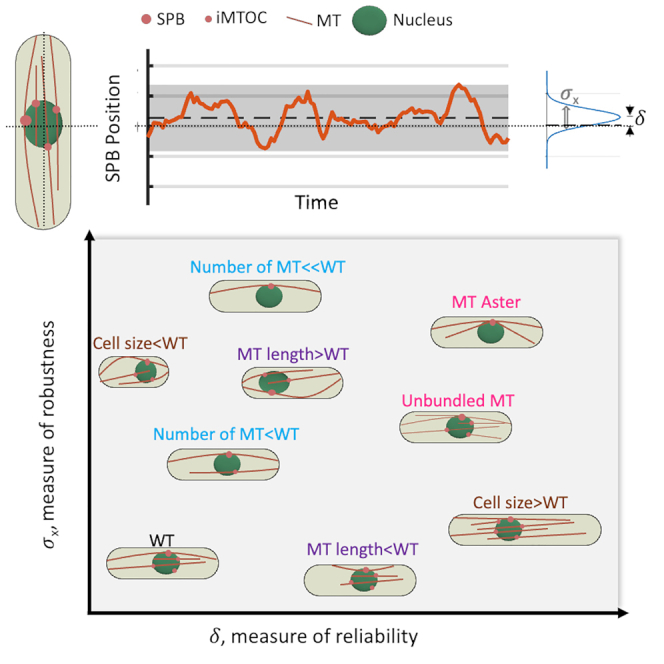

## Introduction

The nucleus and other organelles in eukaryotic cells display precise and reproducible intracellular positioning.^1^^,^^2^^,^^3^ For instance, the nucleus in sea urchin eggs and the mitotic spindle in *Caenorhabditis elegans* localize at the center to within 5% of the cell length.^4^^,^^5^ Reliable positioning is governed by nonequilibrium intracellular forces that arise from self-assembled structures^6^^,^^7^^,^^8^^,^^9^^,^^10^ that are cell spanning, adaptive, and simultaneously pliable and rigid, such as the active cytoskeleton.^11^^,^^12^^,^^13^ However, the quantitative principles underlying the interplay between these force patterns and organelles positioning remain to be elucidated.

Indeed, the positioning of the organelles by fluctuating nonequilibrium forces from the active cytoskeleton appears to be ubiquitous across all eukaryotic life forms. This can arise as a result of pulling forces from actomyosin or motor-microtubule (MT) complexes or pushing forces from MT polymerization-depolymerization.^11^^,^^14^^,^^15^^,^^16^^,^^17^^,^^18^^,^^19^^,^^20^^,^^21^ For instance, nucleus positioning and spindle migration to the cell surface in mouse oocytes is driven by stresses exerted by actin cytoskeleton.^14^^,^^15^^,^^16^^,^^17^^,^^18^ Contractile stresses arising from actomyosin are also seen to be responsible for proper nuclear positioning in mouse fibroblast cells.^18^^,^^22^ In most cells, MT-cytoskeleton-based active forces operate on the cortex either via pushing forces caused by MT polymerization against the cortex or pulling forces applied by anchored, minus-end-directed motors or a combination of these.^19^^,^^20^^,^^21^

Nonequilibrium stresses from both actomyosin contractility and polymerization-depolymerization of MT exhibit strong mechanical fluctuations.^23^^,^^24^^,^^25^^,^^26^^,^^27^ For instance, the growth dynamics of individual MT exhibits dynamical instability,^23^^,^^24^^,^^28^^,^^29^^,^^30^ with a well-defined growth phase before going to “catastrophe” leading to a rapid shrinking phase. Furthermore, the number of MT involved in active force generation is subject to fluctuations, owing to the stochastic dynamics of nucleation and growth. Evidence suggests that the mechanical fluctuations of MTs are sensitive to applied forces^31^^,^^32^^,^^33^; this implies that MT (as indeed actomyosin) are both active *force generat*ors and *force sensors*, aspects that are advantageous for adaptation and robust control.

In this paper, we study the fidelity of nucleus centering in wild type (WT) and genetically perturbed fission yeast (*Schizosaccharomyces pombe*) cells, using a combination of live-cell microscopy and detailed statistical analysis. Fission yeast cells divide by medial fission and produce two daughter cells that are identical in size. Symmetric cell division is contingent on precise localization of the division plane apparatus.^5^^,^^34^^,^^35^^,^^36^^,^^37^^,^^38^^,^^39^^,^^40^^,^^41^^,^^42^^,^^43^^,^^44^ The position of the division septum is determined by the centering of the nucleus at the onset of mitosis,^45^^,^^46^ which in turn is controlled by forces exerted by the dynamic microtubule cytoskeleton.^19^^,^^40^^,^^47^ In the interphase cell, microtubules are organized in 3–4 polar bundles, in which the active plus-ends orient toward the cell tips, and are nucleated from the microtubule-organizing centers (MTOCs) immobilized on the nucleus. Microtubules growing from these bundles can reach the cell tips and exert pushing forces that apply compressive stresses. Consequently, the nucleus feels a force in a direction opposite to the microtubule growth during contact and moves in response. This exclusivity of the nuclear centering force agents makes fission yeast an attractive model system to understand how the patterning and adaptation of the active force machinery influence the reliable and robust centering of the nucleus.

Using high-resolution imaging, we follow the dynamics of the spindle pole body (SPB), a proxy for nuclear movement, in various genetic backgrounds that alter cell length, number of MT bundles, MT organization, and MT growth dynamics. In each of these conditions, we measure the fidelity of centering in terms of *reliability* (δ) - the mean SPB position relative to the geometric center of the cell, and *robustness* ($\sigma_{x}$, $\sigma_{y}$) - the standard deviation of SPB positions in the longitudinal (long.) and transverse (*trans*.) directions. We show that the fidelity of nuclear centering directly affects the ability of the cell to place its septum accurately and divide at the center; the WT condition achieves optimal δ and $\sigma_{x}$, which manifests in low error in septum positioning among the studied perturbations. We identify the key control parameters associated with MT force-patterning and growth dynamics. In the process, we establish that for precise centering of the nucleus, apart from optimum MT number and orientation, the MT length scale (in the milieu of the yeast cell) needs to be matched with the cell length.

Next, we develop a stochastic model for the MT-based nucleus centering, with parameters measured directly from our experiments or estimated from our data using Bayesian inference.^48^ The model recapitulates the high fidelity of the WT; using this, we perform a sensitivity analysis of the parameters that control nuclear centering.

Our model allows us to infer principles of robust and reliable nucleus positioning, leading to several immediate implications that we outline in the discussion.

## Results

### Cell-to-cell variation in reliability and robustness of SPB positioning

**Figure 1:**
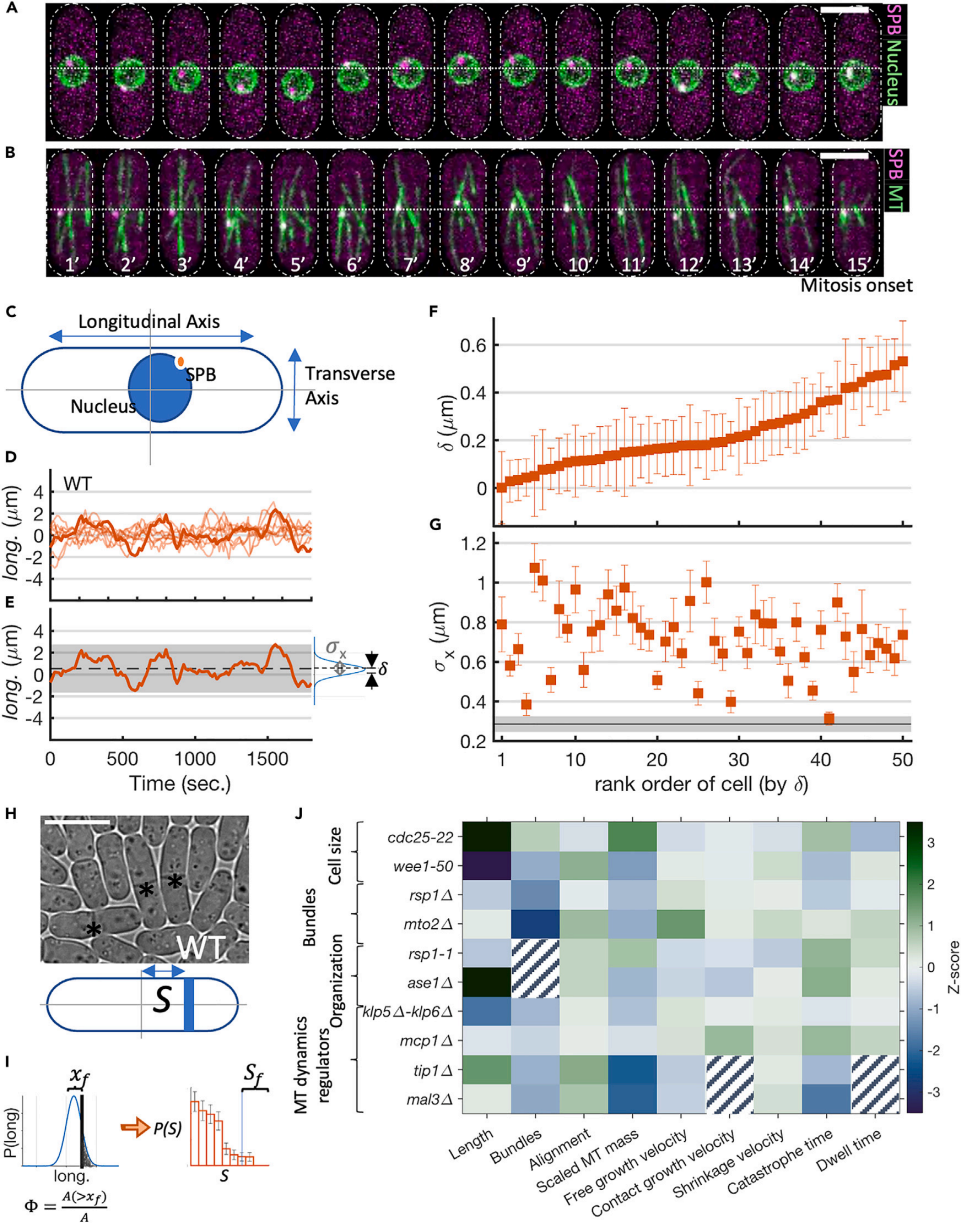
Dynamics of the nucleus and SPB in WT cells (A and B) Time-lapse images of nucleus centering before the mitosis onset. (A) Representative example of the nuclear envelope (Cut11-GFP) and SPB (Sid4-mCherry) fluctuation in WT cell. (B) EnvyGFP-Atb2 Sid4-mCherry tagged strain (WT) showing the dynamics of SPB and MTs. Time-interval 1 min, scale-bar 10 μm. (C) For each cell, we define an internal coordinate system with the origin at the cell centroid and the longitudinal axis aligned with the x axis. By convention, the cells are oriented so that the time-average of longitudinal and transverse displacement of SPB w.r.t. the geometric center of the cell falls in the first quadrant. (D) Example trajectories of longitudinal displacement of SPB. (E) From such trajectories, we estimated the mean longitudinal displacement of SPB from the cell center (δ) and standard deviation in SPB position ($\sigma_{x}$) for each cell. (F and G) Distribution of δ and $\sigma_{x}$ in WT cells sorted by δ. The error bars show standard error, estimated using the moving-block bootstrap method with a block size equal to the autocorrelation time.^51^^,^^52^ The black line (shaded area) in (g) is the mean $\sigma_{x}$ (±SE) in the MT destabilizing drug (MBC)-treated cells. (H) (Top) Examples of septum position in WT cells. (Bottom) The septum position ‘S’ is the distance of the septum from the cell center. (I) To quantify the functional role of δ and $\sigma_{x}$ we define a distance $x_{f}$ from the cell center beyond which the septum localization is considered a *failure*. The fraction of cells ($S_{f}$) whose septum localizes beyond $x_{f}$ depends on δ and $\sigma_{x}$. We calculate the *failure-coefficient* of SPB localization (Φ), by approximating the distribution of SPB as a Gaussian characterized by δ and $\sigma_{x}$, as a readout to likely septum-mislocalization. $\Phi =A\left(>x_{f}\right)/A$, where $A\left(>x_{f}\right)=\int_{x_{f}}^{\infty }p\left(l\right)dl$ and $A=\int_{-\infty }^{\infty }p\left(l\right)dl$. (J) We use systematic perturbations in cell length, the number of MT bundles, MT organization, and MT growth dynamics regulator to understand the role of force patterning in determining SPB positioning. The heatmap shows the difference between various functional attributes between the WT and the mutant quantified using the *Z* score = $=\frac{{\left\langle j\right\rangle }_{mut}-{\left\langle j\right\rangle }_{WT}}{\sigma_{j}^{WT}}$, where ${\left\langle j\right\rangle }_{mut}$ and ${\left\langle j\right\rangle }_{WT}$ represent the mean values for $j^{th}$ feature of mutant and wild type, respectively, and $\sigma_{j}^{WT}$ is the standard deviation observed in the WT strain in the $j^{th}$ feature (hatched blocks denote cases where the features are ill defined).

Since the SPB localizes on the nucleus and is the nucleator of MT, it provides a convenient peg to follow the position of the nucleus.^45^^,^^49^^,^^50^ We monitor the dynamics of SPB both in WT and in genetic variants, using high spatiotemporal resolution live-cell imaging across 20–50 cells (see Figure 1 and STAR Methods). Simultaneously, we monitor aspects of the MT organization and dynamics that control the positioning of the nucleus. This includes the number of MT bundles, their orientation with respect to the long axis of the rod-shaped fission yeast cells, total MT mass, and MT growth dynamics (Figure 1J). The coordinate system used to measure the longitudinal and transverse displacements from the origin is described in Figure 1C. To standardize the observations between WT and the various perturbations, we used a strain with the following fluorescent tags: Sid4-mCherry (SPB) and EnvyGFP-Atb2 (MT). This background enabled the long-term observation of SPB and MTs.

To verify the validity of SPB dynamics as a readout for the nucleus dynamics, we analyze the dynamics of the centroid of the nucleus and SPB in strains with GFP-Cut11 (nuclear envelop) and Sid4-mCherry (SPB) (see Figures 1A and S1). Figures S1A–S1D show the time course of longitudinal displacement of the nucleus centroid and SPB for the several conditions. The SPB shows larger fluctuations than the nucleus, and thus directly inferring the nucleus dynamics from SPB leads to some uncertainty. However, given that the nuclear membrane is only slightly deformable (Figure 1A), the distribution of SPB positions and the nucleus centroid should be strongly correlated (see Figure S1): Convolution of the nucleus centroid with a uniform distribution of points on the surface of a sphere fits well with the distribution of SPB positions (Figures S1A–S1D). Finally, the principal features describing the fidelity of centering, namely the reliability (δ) and the robustness ($\sigma_{x}$) of the nucleus and SPB, show significant correlations (Figures S1E and S1F) with the Pearson correlation of 0.75 (for δ) and 0.78 (for $\sigma_{x}$).

This suggests that the SPB dynamics can be used as a proxy for nucleus localization before the onset of mitosis. Analysis of the time series of the longitudinal and transverse displacements, during a 30 min period, shows them to be statistically independent (Figure S1). Moreover, the time series is also stationary, with the mean and variance measured over a time window, being independent of time (Figure S1). These measured quantities do not show any change as M-phase approaches, suggesting the dynamics is not subject to additional regulation. Consequently, the spatial position of SPB (and nucleus) at the onset of mitosis is determined exclusively by its prior dynamics.

From the time series of the longitudinal positions of the SPB in a given cell, we compute its stationary distribution, from which we extract δ, the mean SPB displacement from the geometric center, and $\sigma_{x}$, the standard deviation of SPB displacement (Figure 1E). δ is a measure of positioning *reliability*; smaller δ implies a more reliable centering. In ∼40% of WT cells, δ lies significantly away from the center, implying that even WT cells have a significant variation in reliability (Figure 1F). On the other hand, $\sigma_{x}$ is a measure of *robustness* in the positioning, and a smaller $\sigma_{x}$ corresponds to a more robustly localized nucleus. In all WT cells, $\sigma_{x}$ is ∼3-fold higher than the fluctuations observed after disintegrating MTs using MBC (Figure 1G); thus, fluctuations arising from active forces makes a significant contribution to *σ*_*x*_. In principle, δ and $\sigma_{x}$ represent two independent objectives for nucleus centering; changes in either lead to higher chances of an off-centered nucleus and, consequently, a high deviation in septum position (S) from the cell center (Figures 1H and 1I).

In what follows, we will discuss changes upon specific genetic perturbations, keeping in mind that the perturbations inevitably change many attributes related to the organization and dynamics of MTs (quantified in Figures 1J and S2–S6, and discussed further in detail)—we will take this into account in making our inferences.

### SPB dynamics is sensitive to cell length

An aspect of cell geometry that is simple to vary is cell length. Since the MT-based pushing machinery that control nucleus positioning, span the entire length of the cell, perturbations of cell length can potentially inform about adaptability and scaling.^53^^,^^54^^,^^55^^,^^56^^,^^57^

**Figure 2:**
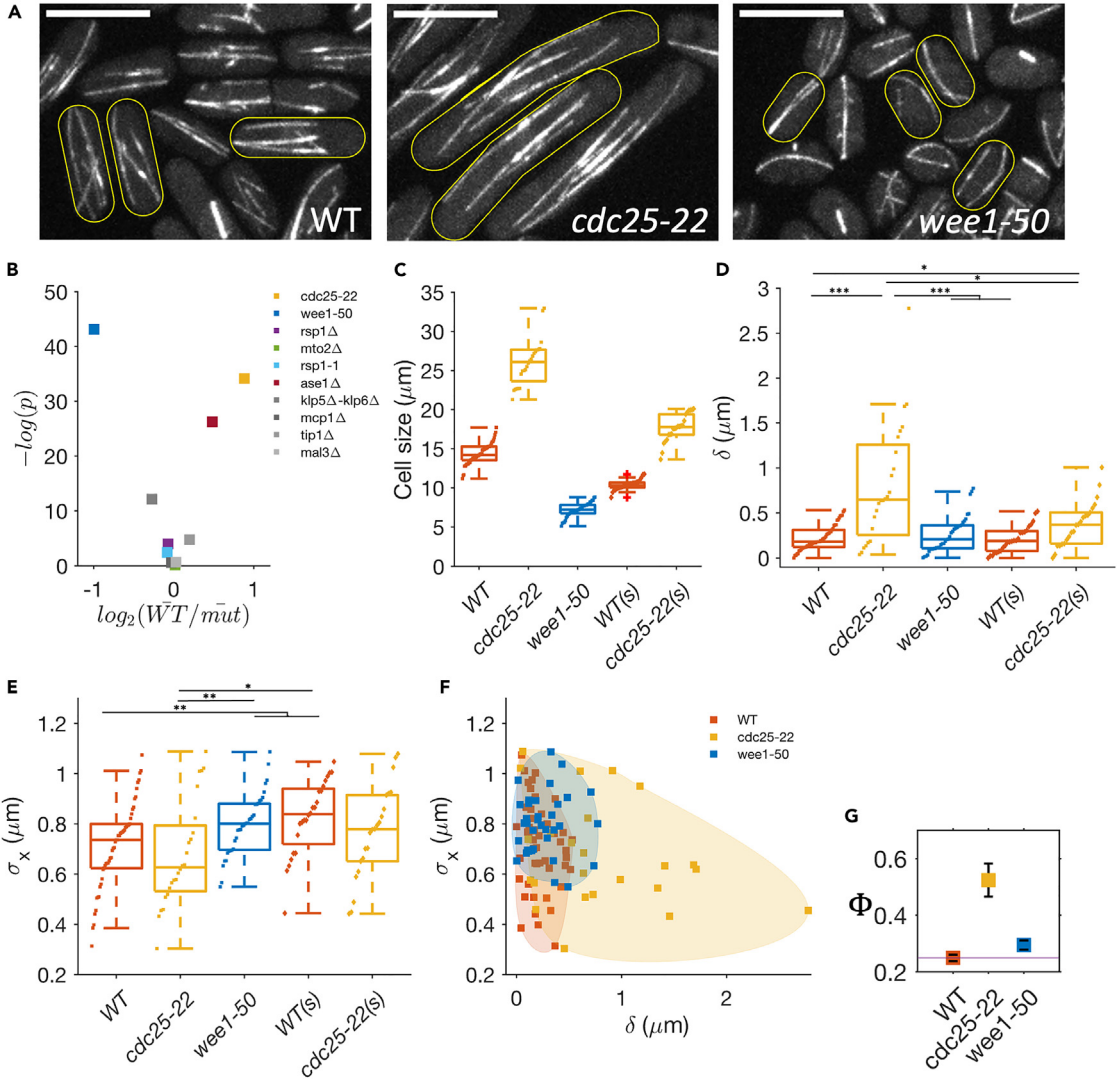
SPB dynamics is sensitive to deviations of cell length from WT (A) MT organization in WT, *cdc25-22* (long), and *wee1-50* (small) cells. Scale bar 10 μm. (B) Scatterplot of statistical significance (p-value) versus magnitude of change (fold change) in cell length w.r.t WT cells in studied strains. *cdc25-22* and *wee1-50* show the most variation in the cell length. (C) Cell length distribution at the onset of mitosis (for WT and *wee1-50*) and in the long *cdc25-22* cells (also Figure S6). (D) The mean longitudinal displacement of SPB from the cell center (δ) for each cell was obtained from SPB dynamics data (see Figure S2). δ increases in the long cells irrespective of the function of Cdc25. (E) Standard deviation in SPB position ($\sigma_{x}$) for each cell. $\sigma_{x}$ increases in short cells. (F) Scatterplot of δ and $\sigma_{x}$ for WT, *cdc25-22*, *wee1-50* cells. A large fraction of *cdc25-22* have δ larger than both WT and *wee1-50* and for a given value of δ small *wee1-50* have higher $\sigma_{x}$ than the WT or *cdc25-22*. (G) For each strain, we calculate the mean Φ as a function of δ and $\sigma_{x}$ as a measure of nucleus mislocalization. (error bars are SE). [∗∗∗ for p ≤ 10^−4^, ∗∗ for p ≤ 0.005, ∗ for p *<* 0.05, Wilcoxon rank-sum test. N = 20–50 cells.].

We utilized the cell cycle mutant *cdc25-22* in which G2-M transition is delayed leading to longer cells; and *wee1-50* mutant strains, which shortens the cell cycle by inducing early G2-M transition, leading to shorter cells.^58^ Together with WT, these strains provide a 4x variation in cell length (Figures 2A–2C).

The basic organization of MT cytoskeleton in these strains is similar—MTs are arranged in bundles, mostly aligned with the long axis of the cell (Figures 2A and S2). However, in the short *wee1-50* cells, MTs are more bent, resulting in a dispersed local MT orientation (Figure S2). The number of MT bundles and the net MT mass per unit length is large in *cdc25-22* cells and small in *wee1-50* cells compared to the WT, suggesting a systematic variation with cell length (Figure S2). Importantly, measurement of the MT growth parameters (see STAR Methods and Figure S7) reveal that the free growth velocity, shrinkage velocity, and growth velocity during contact of MT to cell cortex are very similar to the WT (Figure S2). The catastrophe time, however, shows a slight variation, averaging 115, 148, and 90 s for the WT, *cdc25-22*, and *wee1-50* cells, respectively (Figure S2). Similarly, the mean dwell times are 45 s for the WT, 17 s for *cdc25-22*, and cells 54 s for *wee1-50*. Taken together, these results suggest that cell length perturbations have only a moderate effect on the basic MT organization and intrinsic growth parameters. However, the dynamic parameters that rely on the interaction between MT and cell cortex, e.g. the catastrophe and dwell times,^32^ and MT mass show a dependence on cell length.

We now monitor the SPB dynamics before the onset of mitosis both in the WT and these strains. A visual inspection of the time series of the longitudinal positions immediately reveals that *wee1-50* cells exhibit larger fluctuations than the WT cells, while the *cdc25-22* cells show smaller fluctuations than the WT (Figure S2). In addition, in many *cdc25-22* cells, the mean SPB localization is visibly off-center. Consequently, many cells in the *cdc25-22* strain have large values of δ compared to the WT or *wee1-50* cells, suggesting inadequate reliability of positioning. This difference is independent of Cdc25 function, since in small *cdc25-22* cells, δ decreases (Figure 2D). We see that *wee1-50* cells have significantly large $\sigma_{x}$ in comparison to WT or *cdc25-22* cells (Figure 2E), suggesting a less robust localization of nucleus in small cells. This difference is independent of Wee1 function since small WT cells also have large $\sigma_{x}$ (Figure S2). We conclude that an increase (decrease) in cell length from the typical WT leads to less reliable (less robust) nucleus centering, respectively.

The observations of high $\sigma_{x}$ in the shorter cells are reminiscent of the high fluctuations seen in *in vitro* assays where MT lengths are much longer than the compartment size.^59^^,^^60^ The buckled MTs appear less dynamic and are associated with lower $\sigma_{x}$.^61^

Together, reliability (δ) and robustness ($\sigma_{x}$) are the two independent phenotypic objectives for nucleus position at the onset of mitosis; good performance in these two objectives correspond to low values of both δ and $\sigma_{x}$. We can ask how each cell in these strains fare in the realization of these joint objectives. Owing to underlying cell-to-cell variations, we expect a cloud of points to represent each strain (the color-shaded region in Figure 2F) in an objective space. Large *cdc25-22* cells occupy an expanded space where many cells have larger δ than the WT and *wee1-50* cells. Conversely, small *wee1-50* cells occupy a region where many cells have higher $\sigma_{x}$ than the WT cells. These observations suggest that collective minimization of δ and $\sigma_{x}$ is sensitive to the cell length.

An increase in either δ or $\sigma_{x}$ enhances the chances of mislocalization of the septum. We define the *failure-coefficient* of SPB localization (Φ), that quantifies the likelihood of extreme septum localization events (Figure 1I) beyond a fixed threshold, say the extreme-5% septum localization events in the WT cells. Comparing Φ across the three strains suggests that the chance of mislocalization is the least in the WT cell length (Figure 2G).

### Robustness in nucleus positioning increases with number of MT bundles

**Figure 3:**
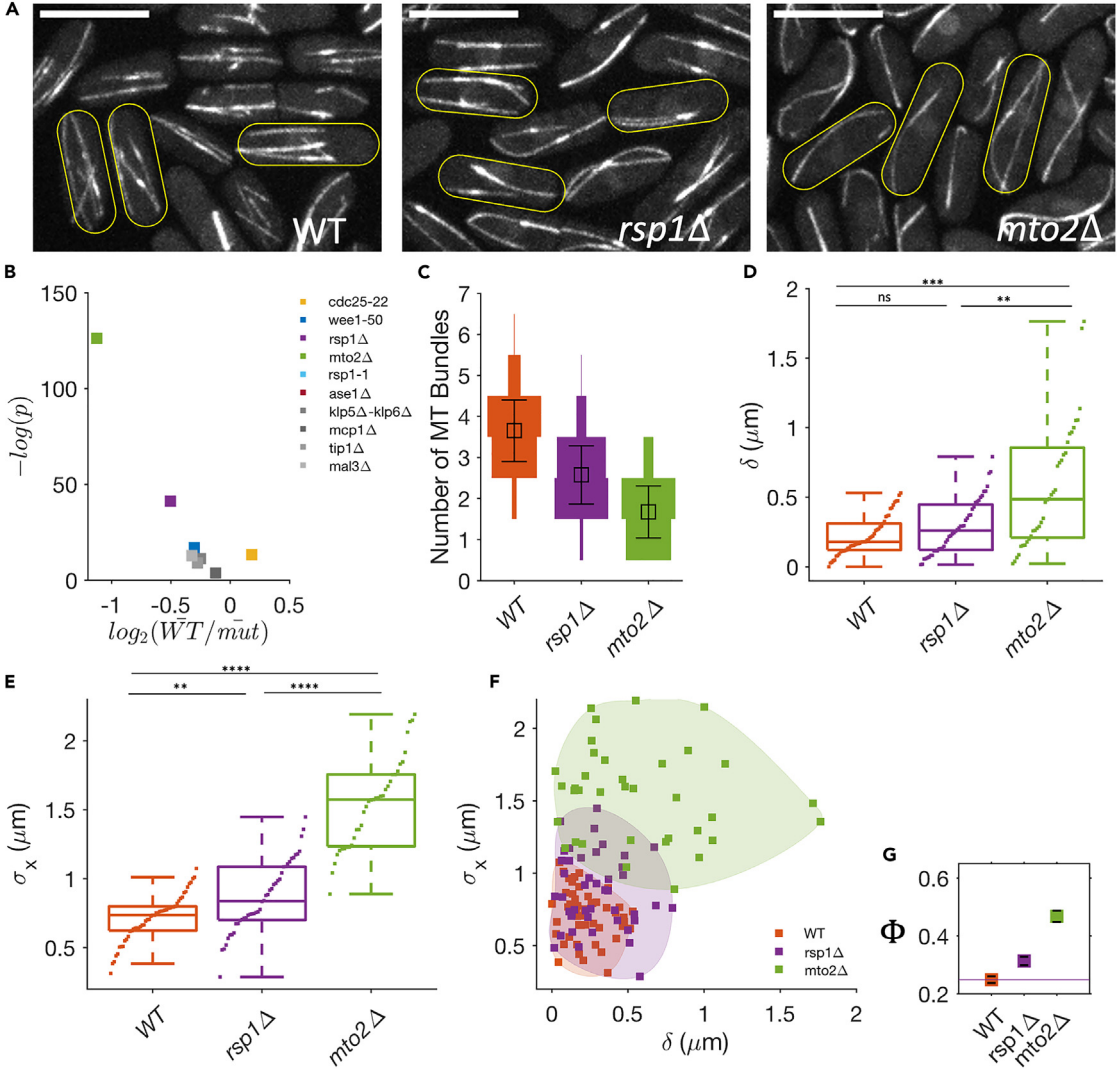
Fidelity of nucleus centering is affected by the number of MT bundles (A) MT organization in WT, *rsp1*Δ, and *mto2*Δ cells. Scale bar = 10 μm. (B) Scatterplot of statistical significance (p-value) versus magnitude of change (fold change) in the number of MT bundles w.r.t WT cells in studied strains. (C) Distribution of the number of MT bundles with a mean equal to 3.7 (WT), 2.5 (*rsp1*Δ), and 1.6 (*mto2*Δ) (error bars are SD) (also Figure S6). (D) Mean longitudinal displacement of SPB from the cell center (δ) for each cell obtained from SPB dynamics data (see Figure S3). (E) Standard deviation in SPB position ($\sigma_{x}$) for each cell. $\sigma_{x}$ increases with a decrease in the number of MT bundles. (F) Scatterplot of δ and $\sigma_{x}$ for WT, *rsp1*Δ, and *mto2*Δ cells. For a given δ,$\sigma_{x}$ follows the trend WT*<rsp1*Δ*<mto2*Δ. (G) The mean Φ as a function of δ and $\sigma_{x}$ as a measure of nucleus mislocalization. (error bars are SE). [∗∗∗∗ for p ≤ 10^−5^, ∗∗∗ for p ≤ 10^−4^, ∗∗ for p ≤ 0.005, ∗ for p *<* 0.05, ns is not significant. Wilcoxon rank-sum test. N = 20–50 cells.].

We now look at how one may systematically vary parameters characterizing MT-based force patterning. Here, we study how variation in MT bundle numbers affects nucleus positioning. WT cells have 3–4 MT bundles which consist of up to 12 filaments arranged in a predominantly anti-parallel manner.^7^^,^^8^^,^^47^^,^^62^ We study deletion mutants where this bundle number can vary—thus *rsp1*Δ has 2–3 and *mto2*Δ has 1–2 MT bundles (Figures 3A–3C). Rsp1 localizes to interphase-MTOCs (iMTOCs), non-SPB MTOCs which are only present during interphase, and is required for the proper functioning of iMTOCs.^63^^,^^64^ Mto2 is a component of the *γ*-tubulin activator complex, and deletion of Mto2 leads to nonfunctioning iMTOCs, which results in SPB being the only active MTOC.^47^^,^^49^^,^^65^ Both these mutants have cell size similar to WT cells at the onset of mitosis (Figure S3).

The *rsp1*Δ and *mto2*Δ cells have very similar MT mass, though significantly reduced in comparison with WT (Figure S3). Not surprisingly, *mto2*Δ cells show a higher fraction of bent MTs (Figure S3). We find that most of the dynamic parameters associated with MT growth are not significantly different from the WT, except for the free MT growth velocities which are elevated in the *mto2*Δ strain (Figure S3; Table S1). High MT growth velocities and similar MT catastrophe times are consistent with the observation that *mto2*Δ cells have longer MTs than WT and *rsp1*Δ cells. Furthermore, having the same MT mass for longer MT suggests that *mto2*Δ has fewer MTs numbers than *rsp1*Δ cells. In conclusion, changes in MT bundles in these strains also changes the MT numbers and one should expect the number of MTs to follow the order WT*>rsp1*Δ*>mto2*Δ.

Next, we analyzed the SPB dynamics in these strains 30 mins before the onset of mitosis (Figure S3). We note that in some *mto2*Δ cells, the SPB movement ceases much before the onset of mitosis. This was observed earlier^49^ and attributed to an SPB-MT bundle detachment phenotype; we therefore exclude these cells from our analysis. As seen in Figure S3F, the SPB in *rsp1*Δ and *mto2*Δ cells appears much more dynamic than in WT cells. We quantified the reliability δ and robustness $\sigma_{x}$ of nucleus positioning for each cell (Figures 3D and 3E). The values of δ are significantly higher in *mto2*Δ cells compared to WT and *rsp1*Δ cells, suggesting that many *mto2*Δ cells have less reliable positioning of the nucleus. However, $\sigma_{x}$ appears to increase with a decrease in the number of MT bundles i.e., WT*<rsp1*Δ*<mto2*Δ. This relationship becomes more evident in the objective space of δ and $\sigma_{x}$ (Figure 3F). Moreover, the transverse fluctuations of the SPB are higher in *mto2*Δ than in WT (see Figure S3). This suggests that having multiple bundles also aids in stabilizing the transverse motion of the nucleus.

In comparison to WT strain, *rsp1*Δ and *mto2*Δ strains show a much larger cell-to-cell variation in objective space (Figure 3F). We estimate the failure-coefficient of SPB localization, Φ, using the values of δ and $\sigma_{x}$ for each condition, and find that it depends on the number of MT bundles. We find that the WT with the larger number of MT bundles has the least Φ. There is no appreciable gain beyond a certain number of MT bundles, suggesting a tradeoff between effectiveness and economy in cellular costs.

### Variation in orientational patterning of MT filaments and bundles

**Figure 4:**
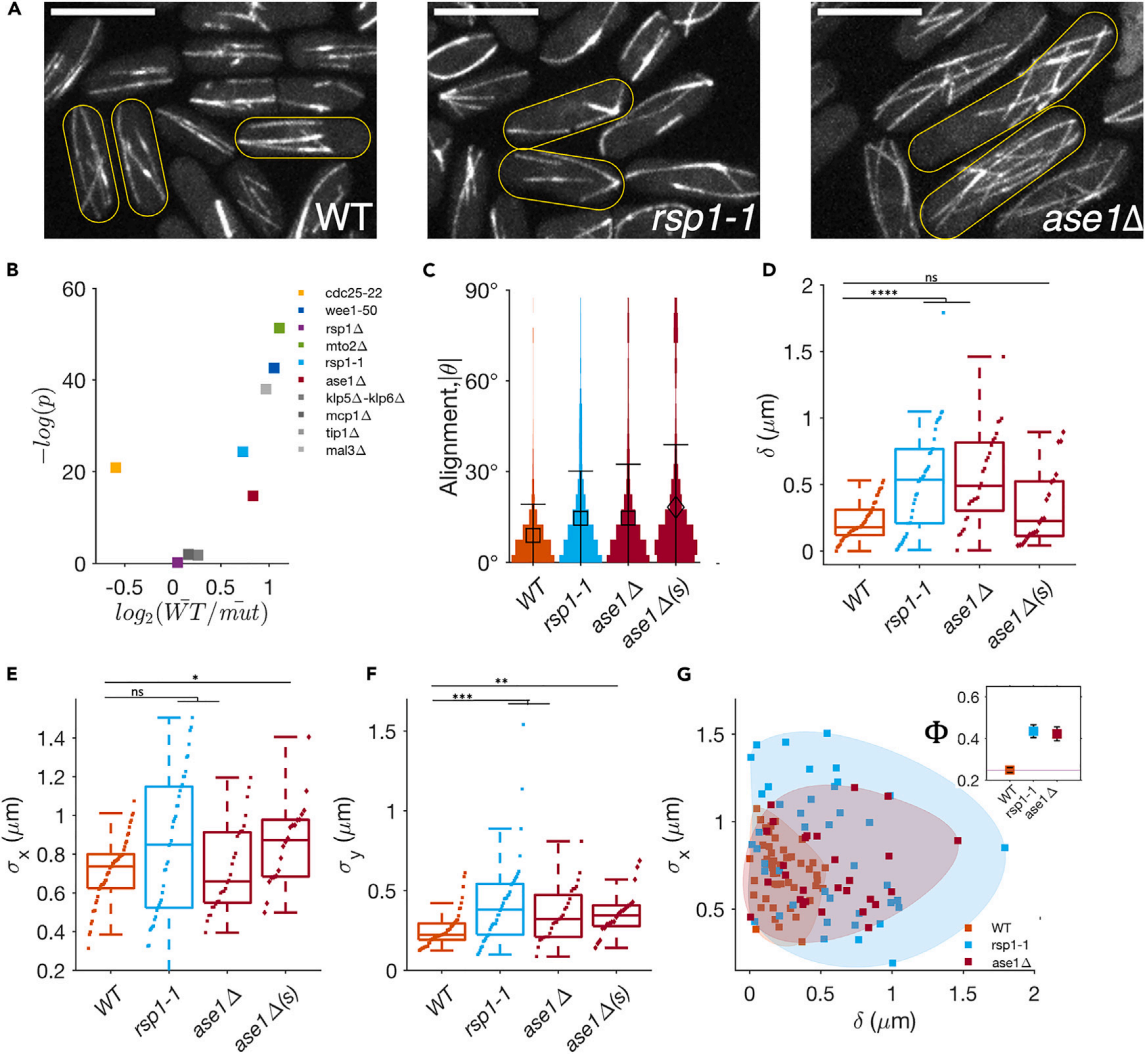
Orientation patterning of MTs affects the fidelity of nucleus centering (A) MT organization in WT, *rsp1-1*, and *ase1*Δ cells. In *rsp1-1* cells, MTs are arranged in an aster-like fashion, whereas in *ase1*Δ cells, MTs are unbundled and disorganized. Scale bar = 10 μm. (B) Scatterplot of statistical significance (p-value) versus magnitude of change (fold change) in the alignment of MT w.r.t WT cells in studied strains. Apart from *rsp1-1* and *ase1*Δ, *wee1-50*, *mto2*Δ, and *mal3*Δ also have a large deviation in MT alignment. However, in *wee1-50* and *mto2*Δ, this large deviation is partly a result of buckled MTs; in the *mal3*Δ cells, mean MT length is small. Moreover, *rsp1-1* and *ase1*Δ have distinct unbundled MT organization. (C) Distribution of alignment of MTs quantified by the absolute local angle |θ| of MT filament relative to the longitudinal axis of the cell. Both *rsp1-1* and *ase1*Δ cells show large angular deviation. The small *ase1*Δ cells show much larger angular deviation than the *ase1*Δ cells. (D) Mean longitudinal displacement of SPB from the cell center (δ) for each cell obtained from SPB dynamics data (see Figure S4). (E) Standard deviation in SPB position ($\sigma_{x}$) for each cell. (F) Transverse fluctuations of SPB ($\sigma_{y}$) in WT, *rsp1-1*, *ase1*Δ, and small *ase1*Δ cells. (G) Scatterplot of δ and $\sigma_{x}$ for WT, *rsp1-1*, and *ase1*Δ cells. Both *rsp1-1* and *ase1*Δ occupy a wider region in the objective space. Inset: The mean Φ as a function of δ and $\sigma_{x}$ as a measure of nucleus mislocalization. (error bars are SE). [∗ ∗ ∗∗ for p ≤ 10^−5^, ∗ ∗ ∗ for p ≤ 10^−4^, ∗∗ for p ≤ 0.005, ∗ for p *<* 0.05, ns is not significant. Wilcoxon rank-sum test. N = 20–50 cells.].

As stated, the MT in WT cells is organized as linear bundles aligned along the long axis of the cell. In contrast, *rsp1-1* mutant cells have MTs that have an aster-like organization, and MT crosslinker, Ase1, deletion strain (*ase1*Δ) has disorganized and non-bundled MTs (Figure 4A).^63^^,^^66^ While *rsp1-1* cells have similar cell length as WT, in our experiments, the typical length of *ase1*Δ cells is longer than WT at the onset of mitosis; therefore we also analyze small *ase1*Δ cells (*ase1*Δ(s)) (Figure S4). The mean scaled MT mass in *rsp1-1* (*ase1*Δ) cells is slightly higher (lower) than in WT cells (Figure S4). The orientation distribution of MT filaments is much broader in *rsp1-1* and *ase1*Δ cells (Figures 4B and 4C) than in WT, with many filaments showing large angular deviations from the long axis.

Despite this, most MT growth parameters in these strains are very similar to the WT (Figure S4). The only significant difference is in catastrophe time, which is higher in both *rsp1-1* (154 s) and *ase1*Δ (157 s) strains in comparison to WT (115 s) (see Table S1).

Figure S4E shows typical SPB trajectories in these cells during the 30 min interval before the onset of mitosis. In some *rsp1-1* cells, the SPB pauses for an extended period near the pole. These typically happen when the MTs are in an aster-like organization.

In *ase1*Δ cells, the mean position of the SPB is displaced away from the cell center. Analysis of δ and $\sigma_{x}$ for *rsp1-1* and *ase1*Δ cells (Figures 4D and 4E) shows that δ is more widely distributed, and that the mean δ is significantly higher than in the WT. On the other hand, $\sigma_{x}$ in *rsp1-1* and *ase1*Δ cells are not significantly different from WT cells. Since *ase1*Δ cells are typically longer than the WT cells, we also study the dynamics of SPB positioning in small *ase1*Δ cells (marked *ase1*Δ(s), also see Figure S4). In small *ase1*Δ cells, while the values of δ are similar to WT cells owing to the MTs making contact with cell tips, $\sigma_{x}$ is higher. Moreover, *rsp1-1* and *ase1*Δ cells displayed larger fluctuations in the transverse displacements of SPB (Figure 4F). The higher fluctuations in the transverse (and longitudinal for *ase1*Δ(s)) direction can be attributed to the large angular deviation of MTs which make prolonged contact with the cylindrical side of cells (away from the cell tips). This results in pushing forces having large angular variations, very different from the coaligned pushing forces in the WT.

In the objective space of δ and $\sigma_{x}$, both *rsp1-1* and *ase1*Δ strain show large cell-to-cell variation, and the failure-coefficient of SPB localization (Φ) increases in both of these strain (Figure 4G). Thus, having linear bundles of MT aligned along the long axis (as in WT) leads to a more reliable and robust nucleus centering.

### Optimal centering of nucleus requires favorable MT growth dynamics

**Figure 5:**
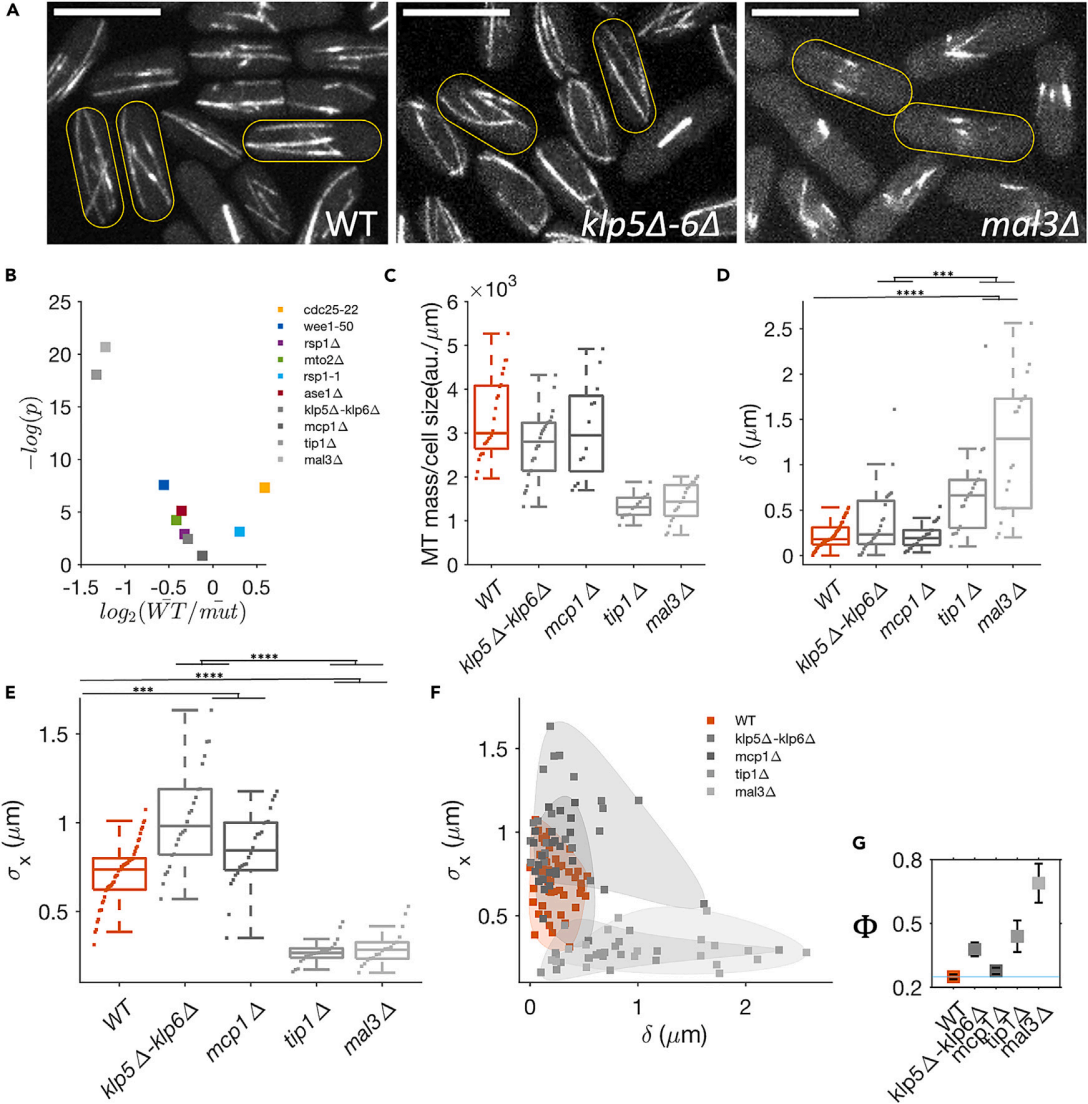
Optimal centering of the nucleus requires favorable MT growth dynamics (A) MT organization in MT growth dynamics impaired mutants (also Figure S5). Scale bar 10 μm. (B) Scatterplot of statistical significance (p-value) versus magnitude of change (fold change) in the scaled MT mass w.r.t WT cells in studied strains. *mal3*Δ and *tip1*Δ (both are MT growth factors) have low MT mass. However, we also observed a reduction in MT mass in *klp5*Δ-*klp6*Δ. (C) MT mass, measured from the intensity of MT fluorescence, scaled with cell length (also Figure S6). (D) Mean longitudinal displacement of SPB from the cell center (δ) for each cell obtained from SPB dynamics data (see Figure S5). (E) Standard deviation in SPB position from the cell center ($\sigma_{x}$) for each cell. (F) Scatterplot of δ and $\sigma_{x}$. The *mal3*Δ and *tip1*Δ strains, with attenuated MT growth, have large δ in the majority of the cells. In *klp5*Δ-*klp6*Δ and *mcp1*Δ strains, many cells have much higher $\sigma_{x}$ than the WT. (G) The mean Φ as a function of δ and $\sigma_{x}$ as a measure of nucleus mislocalization. (error bars are SE). [∗∗∗∗ for p ≤ 10^−5^, ∗∗∗ for p ≤ 10^−4^, ∗∗ for p ≤ 0.005, ∗ for p *<* 0.05, ns is not significant. Wilcoxon rank-sum test. N = 20–50 cells.].

Finally, we systematically studied how variation in the MT growth dynamics affects nucleus centering using the mutants, (i) a deletion strain of Mal3 (*mal3*Δ), an EB1 homolog; (ii) a deletion strain of Tip1 (*tip1*Δ), a CLIP-170 homolog (both are MT growth factors); (iii) a deletion strain of Klp5 and Klp6 (*klp5*Δ-*klp6*Δ), kinesin-8 proteins; and (iv) an Mcp1 deletion strain (*mcp1*Δ), an MT-binding protein (both are MT catastrophe factors)^67^^,^^68^^,^^69^^,^^70^^,^^71^ (Figures 5A and S5). We find that *klp5*Δ-*klp6*Δ cells are shorter and *tip1*Δ cells are longer at mitosis compared to WT (Figure S5). While the MT mass relative to cell length is reduced in *tip1*Δ and *mal3*Δ strains as expected,^24^^,^^67^^,^^72^ we also observe a significant reduction in MT mass in *klp5*Δ-*klp6*Δ strain. This may reflect a decrease in the nucleation activity consistent with the *in vitro* observation that Klp5-Klp6 acts as a nucleation factor^73^ (Figures 5B and 5C). MT orientation in *klp5*Δ-*klp6*Δ and *mcp1*Δ cells is similar to the WT cells (Figure S5). However, in *tip1*Δ and *mal3*Δ cells, the MTs are less aligned along the cell axis, highlighting the significance of long MTs.^40^^,^^74^

The MT growth dynamics in these strains has the following features (Figure S5): Both MT growth and shrinkage velocities are very similar in these strains. The growth velocities of MT during contact with cell tip are also similar in WT, *klp5*Δ-*klp6*Δ, and *mcp1*Δ cells. However, MT undergoes catastrophe much before reaching the cell tip in *mal3*Δ and *tip1*Δ cells, consequently showing higher catastrophe frequency. In our experimental sampling, we do not see much difference between MT catastrophe frequency in WT and *klp5*Δ-*klp6*Δ and *mcp1*Δ cells. However, for *mcp1*Δ cells, we do notice a wider distribution of both MT catastrophe time and dwell times.

Next, we characterized the reliability and robustness of the nucleus centering in these cells (Figures 5D and 5E). Both, *klp5*Δ-*klp6*Δ and *mcp1*Δ cells show higher fluctuations in SPB dynamics compared to WT. In contrast, *tip1*Δ and *mal3*Δ cells, in which the MT catastrophe rate is higher thus leading to shorter MTs, show suppressed SPB dynamics away from the cell center (Figure S5). This leads to high value of δ (Figure 5D), similar to the long *cdc25-22* cells. However, both *klp5*Δ-*klp6*Δ and *mcp1*Δ cells show much higher $\sigma_{x}$ (Figures S5 and 5E). Earlier, high $\sigma_{x}$ in *klp5*Δ-*klp6*Δ has been attributed to the lack of enhanced catastrophe at cell ends;^50^ however, we propose that a reduction in MT might also contribute to this phenomenon. Cumulative analysis of δ and $\sigma_{x}$ in the objective space (Figure 5F) shows that *mal3*Δ and *tip1*Δ prominently occupy a space to the right of the WT with smaller $\sigma_{x}$ and *klp5*Δ-*klp6*Δ and *mcp1*Δ cells occupy space above the WT with small δ. The failure-coefficient Φ of SPB localization (Figure 5G) shows that the altered MT growth dynamics increases the chance of nucleus mispositioning.

### Precise nucleus centering is key to medial positioning of division plane

**Figure 6:**
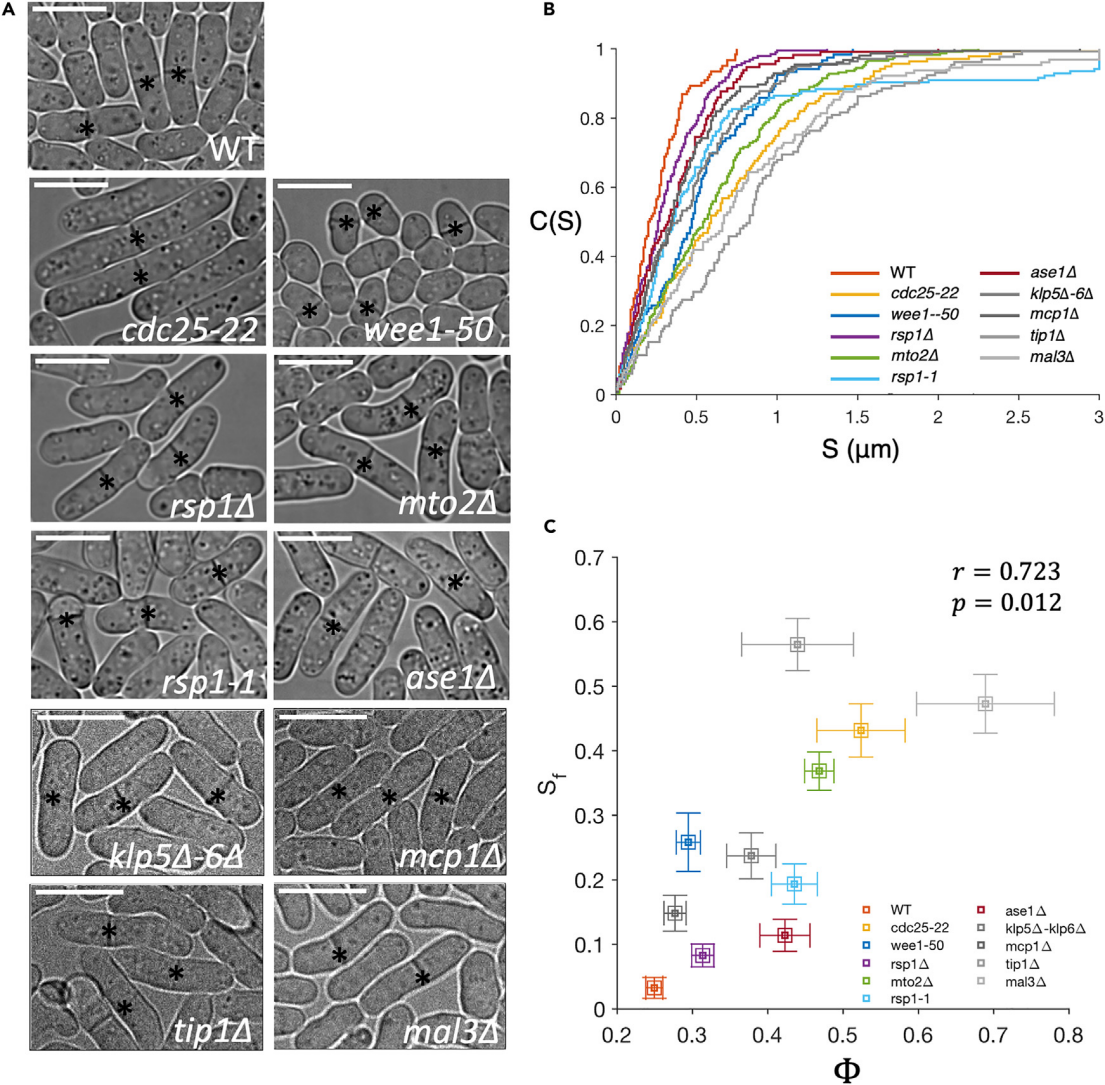
Proper positioning of the septum is contingent on nucleus centering (A) Panel shows examples of septum location in different strains. Scale bars 10 *μm*. Asterisks (∗) mark the septa. (B) Cumulative distributions of S for various stains (also Figure S6 for p values between these strains). (C) Fraction of cells with failed septum localization ($S_{f}$) correlates with mean failure-coefficient of SPB localization Φ for the different strains, indicating the relevance of the combined optimization of δ and $\sigma_{x}$. Error bars represent standard error. The error bar on $S_{f}$ is estimated using 100 boot-strapped replicas (N = 120–200 cells).

A crucial function of nuclear positioning in fission yeast at the onset of mitosis is to define the site of septum formation. We have shown that cell length, MT bundle numbers, MT architecture, and MT growth dynamics influence both reliability and robustness of nucleus centering. We next ask how do these traits impact the septum position. To this end, we observed the septum position in the studied strains at 35°C. (Note that *cdc25-22* cells do not commit to mitosis at 35°C. However, we found that *cdc25-22* cells growing at 35°C immediately go to mitosis on changing the temperature to 25°C, see STAR Methods). The off-center septum is visible in many strains. We quantify the error in septum positioning by measuring the distance of the septum from the cell center (S) (Figure 1H). Figure 6B shows the cumulative distribution of S in these strains. It is immediately clear that all mutants show a much broader distribution of septum positions than WT. Both, high δ or high $\sigma_{x}$ of the SPB-position get mapped onto a broad distribution of S. For example, the *wee1-50* and *mcp1*Δ strains have higher $\sigma_{x}$ than WT, and *ase1*Δ has higher δ than WT, even though the $\sigma_{x}$ is comparable—all these strains exhibit a much broader S distribution.

Based on these observations, we expect that $S_{f}$, the failed septum localization (fraction S that lies beyond $x_{f}$), should be strongly correlated with Φ (fraction of cells with failed SPB localization), as Φ is a function of both δ and $\sigma_{x}$ (Figure 6C). Pearson correlation statistics between the mean Φ and $S_{f}$ shows a correlation of 0.72 (p *<* 0*.*05). We note that some of the mutants explored here are also known to show some degree of morphological variations; e.g., *tip1*Δ, *mal3*Δ have noticeably bent cells in their population. These variations have been linked to the role MTs play in the disposition of polarity factors.^40^^,^^75^^,^^76^ Given these complexities, we contend that the ∼72% variation in $S_{f}$ explained by Φ reflects the high influence of optimal nucleus centering on the septum position.

### Stochastic model of MT-driven nucleus centering recapitulates the optimality of the WT

We next develop a stochastic model to explain how the underlying MT force patterning may lead to an optimum nucleus centering. We have seen that the position of the nucleus in the interphase cell is controlled by forces exerted by the dynamic MTs which nucleate at the nucleus and are organized in a finite number of bundles. MTs polymerizing from these bundles can reach the cell tips and exert pushing forces, undergo dynamical instability, and shrink. Consequently, the nucleus feels a stochastic force in a direction opposite to the MT growth during the contact phase and moves in response.

**Figure 7:**
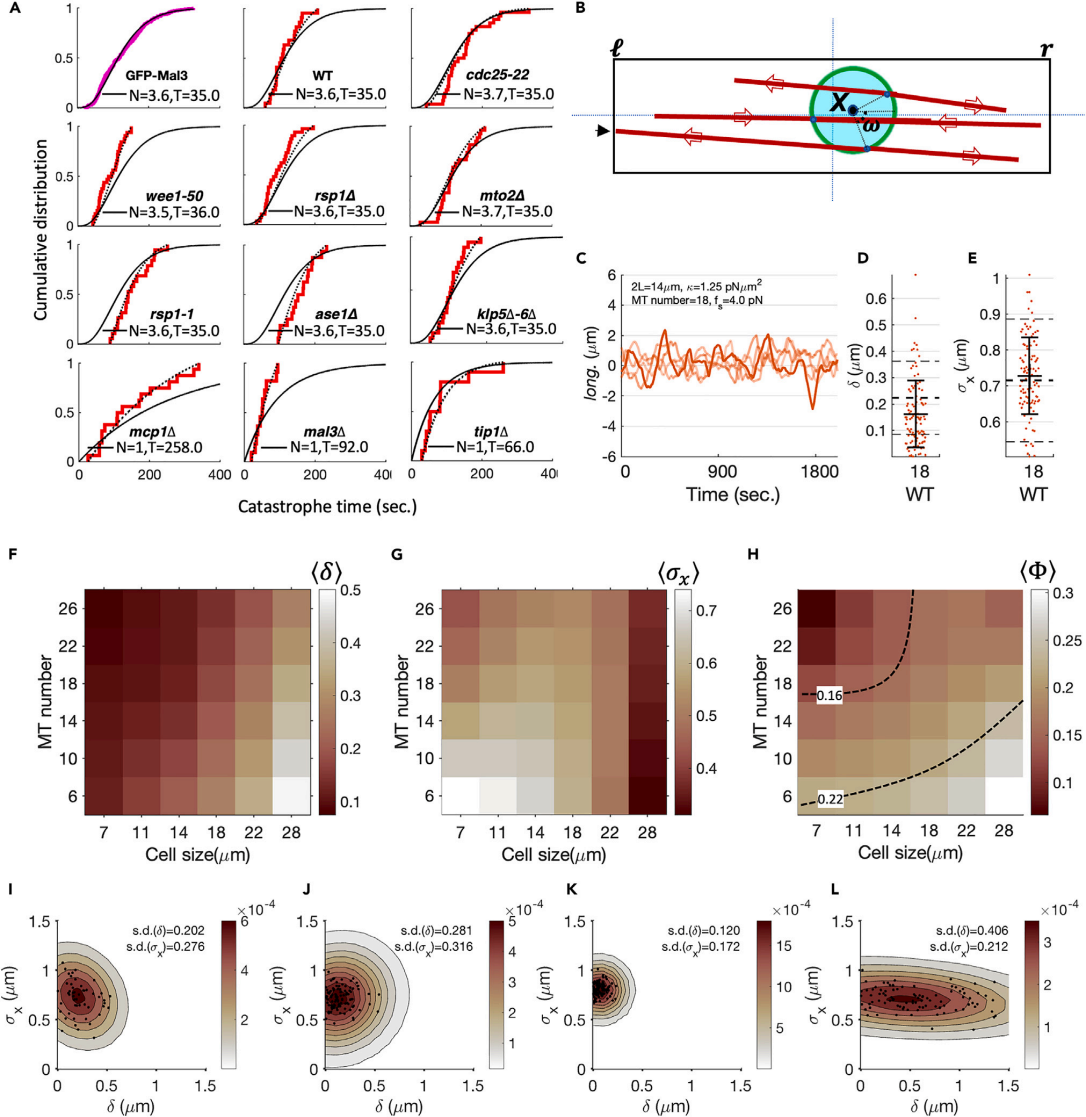
Stochastic model recapitulates the optimality of WT (A) (Top-left panel) Cumulative distribution of $\tau_{cat}$ (magenta) obtained from Mal3 strain. A gamma distribution gives the best fit (black curve) for the distribution of $\tau_{cat}$. (Other panels) Parameters obtained using the Bayesian analysis of the $\tau_{cat}$ obtained for various strain utilizing EnvyGFP-Atb2 Sid4-mCherry background. The red line shows the cumulative distribution from experiment. The black curve shows the full distribution inferred using Bayesian analysis. The dotted curve is truncated distribution obtained using the estimated parameters (black curve) with truncation at the minimum and maximum of experimentally observed $\tau_{cat}$ for respective strains. (B) Schematic depicting elements of the theoretical model for MT-driven nucleus centering. The MTs originate from MTOCs (blue dots) mounted on the periphery of a rigid nucleus. The direction of the MT growth is represented by red arrows. The pushing forces arising at the MT cell wall contacts are represented by black arrow (see main text and supplemental information for a detail description). (C) Example trajectories of SPB obtained from simulations. (D and E) Statistics of δ (D) and $\sigma_{x}$ (E) obtained using the parameters listed in (C). The dotted lines are experimental values of means (wide-lines) and standard deviation (thin-lines) for respective statistics. Error bars are standard deviation. (F and G) Discrete contour-plots showing the systematic effect of variation in cell length and number of MTs on δ (F) and $\sigma_{x}$ (G). Color-bar scale is in μm. The rest of the parameter-set are same as in (C). (H) A discrete contour-plot showing the effect of variation in cell length and number of MTs on failure-coefficient of SPB localization Φ. The dashed curves are iso-contours for two values of Φ. (I–L) Bivariate probability distribution of δ and $\sigma_{x}$. Scatterplots show cell-to-cell variations. The contour shows probability distribution estimated by fitting a 2D-Gaussian after convolving the data points with a symmetric Gaussian kernel. Color bar scale shows probability density. (I) Experimental data from WT strain showing cell-to-cell variation in $\sigma_{x}$ are larger than δ suggesting that the control on δ is *stiffer* than on $\sigma_{x}$. (J) Simulations with parameters listed in (C) with the cell length following the experimentally observed cell length variation. (K) Simulations with perfect correlation between left (*l*) and right (*r*) MT force generators, in terms of their local orientations, and iMTOC localization on the nucleus. (L) Simulations with uncorrelated left-right MTs orientation.

This is summarized in the following dynamical equations in terms of nonequilibrium forces and torques on the nucleus:

(Equation 1) $$\zeta {\left(t\right)}^{T}\dot{X}=\sum_{i=1}^{N_{r}}f_{i}^{r}+\sum_{i=1}^{N_{l}}f_{i}^{l}\text{,}$$

and

(Equation 2) $$\zeta {\left(t\right)}^{R}\dot{\omega }=\sum_{i=1}^{N_{r}}T_{i}^{r}+\sum_{i=1}^{N_{l}}T_{i}^{l}\text{,}$$

where X and ω are the position of the centroid of the nucleus (taken as a rigid sphere) and angle between the longitudinal axis of the cell and the vector connecting the centroid of the nucleus to SPB, respectively. The effective translational (T) and rotational (R) drag matrices $\zeta {\left(t\right)}^{T/R}$ are a combination of the drag coefficients of the nucleus and MT bundles. The force $f^{r/l}$ and the torque $T^{r/l}$ are applied by $N^{r/l}$ MTs directed toward the right (r) and left (l) cell tips (Figure 7B, see detailed description in STAR Methods). The nucleus experiences a pushing force only when the MTs are in contact with the right or left cell boundary,

(Equation 3) $$f=\left\{ \begin{array}{cc} f_{p} & iff_{p}\leq f_{e}\\ f_{e}\text{,} & otherwise \end{array}\right.$$

where $f_{p}$ is the MT polymerization-driven pushing force described by the force-velocity relationship: $f_{s}\left(1-V_{+}^{f}/V_{+}\right)$, where $f_{s}$ is the stall force and $V_{+}$ and $V_{+}^{f}$ is the growth velocity of MT during the free growth and dwell phase, and $f_{e}$ is the critical Euler buckling force of MTs, which depends on the MTs flexural rigidity κ (Table S2). Contact with the cell tips lasts only until the onset of catastrophe, wherein the MTs shrink rapidly and exert zero force until it recovers, grows, and reaches the cell tip again. The catastrophe times $\tau_{cat}$ and their recovery are stochastic, and we measure their statistical distribution. In our analysis, we include a force-dependent reduction in catastrophe time via the relation $\tau_{cat}\left(V_{+}^{f}\right)=\tau_{o}+\frac{\tau_{cat}\left(V_{+}\right)-\tau_{o}}{V_{+}}V_{+}^{f}$, where $\tau_{o}$ is the mean catastrophe time at $V_{+}=0$.^32^^,^^77^ We measure these growth (and shrinkage) velocities in the WT and the different mutant strains, and find them similar (Figures S2–S5, Table S1).

We find that the experimentally determined $\tau_{cat}$ across strains is best modeled by a gamma distribution $P\left(\tau_{cat}|N,T\right)=\frac{\tau_{cat}^{N-1}\;\exp\left(-\tau_{cat}/T\right)}{T^{N}\Gamma \left(N\right)}$, characterized by two parameters - a step parameter (N) and timescale parameter (T), where Γ(N) is the usual Gamma function^78^^,^^79^ (Figure 7A). We utilize the framework of Bayesian inference to deduce the parameters of MT catastrophe distribution in different strains (see STAR Methods). Bayes rule assigns the posterior probability of a set of model parameters (θ={N,T}) given the observations ($O=\tau_{cat}$) by,^48^

(Equation 4) $$p(\theta |O)=\frac{p\left(O|\theta \right)\;p\left(\theta \right)}{p\left(O\right)}\text{,}$$

where p(θ) is the prior distribution of the model parameters, p(O|θ) is the likelihood function of the observations O given the parameters θ, and p(O)=∫p(O|θ)p(θ)dθ is the marginal likelihood distribution of the observations (see STAR Methods). To estimate an informative prior, we obtain a likelihood distribution from the rather abundant and reliable data on the Mal3 strain (Figure S8, also STAR Methods) and combine it with a flat (uniform) prior (see STAR Methods for details). We then use this informative prior to obtain the distribution of parameters that best describes the observations $\tau_{cat}$ in each strain. As a result, we find that with the exception of *mcp1*Δ, *mal3*Δ, and *tip1*Δ mutant strains (all regulators of MT growth dynamics), all other strains studied have very similar parameters associated with the $\tau_{cat}$ distribution (Figure 7A). In addition, a truncated distribution obtained by restricting the full distribution at experimentally observed minimum and maximum values of $\tau_{cat}$ suffices to explain the observed empirical distribution.

We now proceed to estimate the remaining MT parameters, the stall force $f_{s}$, and the flexural rigidity κ (the growth and shrinkage velocities have been measured directly from experiments). We do this by fitting the simulated distributions of δ and $\sigma_{x}$ from the model Equations 1 and 2 (see STAR Methods) to the distribution obtained from the WT data (see Figure S10). This gives a value of $\kappa \approx 1.25pN\mu m^{2}$, which goes to set $f_{e}$. This value of κ falls in the lower end of the range of MT stiffness obtained in *in vitro* measurements,^80^ and is consistent with the bent and curved MTs observed in the WT as well as the small cell strains, such as the *wee1-50*. The fit however does not fix $f_{s}$ as along as $f_{s}>1pN$. If we choose $f_{s}$ ranging from 4 to 6 pN, then we find that the mean catastrophe times, dwell-times of MTs growth dynamics, and auto-correlation function of nucleus also matches with the experimental observations. For this range of values of *f*_*s*_, we are indeed in the regime $f_{s}>f_{e}$ where MT buckles before the MT growth stalls.

Having established the kinetic parameters of our model that take very similar values in the different strains (Table S2), we are now in a position to compare the nuclear trajectories and the statics of nuclear positioning, δ and $\sigma_{x}$, from the model with those obtained from the experiments (Figures 7C–7E). The cell-strain-dependent features that we vary in the model include the MT number $N^{r/l}$, the orientation pattern of the filaments, and the cell length (Figures 7F and 7G).

Our stochastic model recapitulates the observation that δ and $\sigma_{x}$ show opposite trends upon changing MT number and orientation and cell length away from the WT. For instance, from our model, we find that independent of MT number, δ increases with an increase in the cell length away from WT. This is consistent with our experimental observations. Moreover, δ decreases as a function of increasing MT number, keeping everything else fixed.

On the other hand, the model shows that $\sigma_{x}$ decreases with an increase in cell size away from WT and decreases with increasing MT number, again consistent with experimental observations. For small MT number, the relative fluctuations of the force bearing MTs to the left and right is large, leading to an increase in $\sigma_{x}$.^61^^,^^81^ Note that the MT number affects both the MT polymerization-induced forces and the effective drag on the nucleus.

Our stochastic model recapitulates the observation that it is the combination of δ and $\sigma_{x}$ that is functionally relevant for the position of the nucleus at the onset of mitosis. As before, this can be expressed by the mis-centered fraction of SPB localization Φ (Figure 7H), with the following results: (i) Keeping cell length fixed (say, = 14 *μ*m), the value of Φ decreases monotonically as the MT number increases before saturating at high MT number. This suggests that a further increase in MT numbers does not translate into significant decrease in Φ, consistent with experimental findings (ii) Keeping MT number fixed (say, = 18), the value of Φ increases beyond a certain cell length = 14 *μ*m (Figure 7H). (iii) Along a fixed contour of Φ (say, Φ = 0*.*16 dashed curve in Figure 7H), variation of the cell length away from WT (= 14 *μ*m) is compensated by larger MT numbers.

The anisotropy in the joint distribution of δ and $\sigma_{x}$ provides on the *control* of nucleus positioning. The bivariate distribution width is much broader in the $\sigma_{x}$ direction than the δ direction (Figure 7I), implying that control on δ is *stiffer* than $\sigma_{x}$.^82^^,^^83^ We find that the reliability δ is predominantly controlled by the strong correlation between the left and right-oriented MT force generators and correlations in the localization of iMTOCs on the nucleus. On the other hand, the robustness $\sigma_{x}$ is controlled by cell length, MT length scale, and number of MT bundles. Figure 7J shows a comparison of the distribution of δ and $\sigma_{x}$ between the experimental WT and the stochastic model where the cell lengths take values over the experimental range. In addition, in our model, we can vary the left/right correlation of the MT force generators. We find that a perfect correlation reduces the width of the variation along δ, while a weaker correlation increases it substantially (Figures 7K and 7L).

## Discussion

In this paper, we have used live-cell imaging to study the fidelity of nucleus centering (and hence of the septum) in WT and genetically perturbed fission yeast cells in terms of two phenotypic objectives for nucleus position at the onset of mitosis, namely reliability (δ) and robustness ($\sigma_{x}$). The genetic perturbations that we have studied go to change cell length, MT number, and their orientational patterning and MT dynamics. In conjunction, we have analyzed a stochastic model that describes the nucleus centering in terms of MT polymerization-induced forces. Extracting the parameters of this model from experiments, we were able to compute the corresponding δ and $\sigma_{x}$, as one varied the MT number, orientation, and cell length. Both experiment and theory show that high fidelity in nucleus centering is a consequence of the collective optimization of δ and $\sigma_{x}$ in nucleus positioning, which is akin to finding Pareto-optimal conditions.^84^^,^^85^^,^^86^^,^^87^ Our analysis has interesting implications that we list below.

### Optimal MT length and implications for cell length selection

The first implication is that the length scale of MTs growth needs to be comparable with the cell length. MT length-scale is determined by the distribution of catastrophe time along with the growth and shrinkage velocities. We showed that catastrophe time distribution across many perturbations can be modeled by a gamma distribution. The surprising finding is that the parameters of the distribution do not show significant difference, suggesting that MT dynamics and consequently the MT length are unaltered by the perturbations we have imposed on the cells. This is likely because the perturbations do not affect the MT (dis)assembly machinery; specific MT regulator deletion strains do produce deviations in the MT catastrophe times distribution. While it is possible that this distribution gets altered because of force-enhanced catastrophe,^32^ this appears to be small. This optimality in nucleus centering in the WT, as a consequence of the matching of the MT length (in the milieu of the yeast cell) and the WT cell length, might have implications for cell size selection in fission yeast.

### Proper MT number and orientation for optimal force patterning

The proper centering of the nucleus is contingent on proper nonequilibrium force patterning that is generated by MT-polymerizing forces. Thus, the force patterning is described by the orientation of the MTs, their arrangement in bundles, and the number of MT bundles. In fission yeast, this is achieved by a noncentrosomal MT organization with MT arranged in a small number of bundles emanating from the nucleus. Our study shows that the optimal orientation of MTs is when they form bundles coaligned with the long axis of the cell. Deviation from coalignment leads to aberrant centering. Any change from this arrangement increases δ and $\sigma_{x}$. Organizing MTs in coaligned 3–4 bundles distributed around the nucleus diminishes transverse fluctuations $\sigma_{y}$ driven by the torque.

The performance of the two phenotypic objectives improves with an increase in coaligned MTs;^12^^,^^61^^,^^81^ however beyond a certain number, there is no significant gain. Beyond approximately *N* = 22 MT, the improvement if any is marginal, suggesting as in any biological control system, a tradeoff between effectiveness and economy.

### Combined optimization of δ and $\sigma_{x}$ implies feedback and adaptation of microtubule pushing forces

The optimality of nucleus position fluctuations, codified by δ and $\sigma_{x}$, is a natural consequence of the fluctuating MT polymerization forces. The opposing MT-polymerization-based pushing forces can be viewed as a stochastic control on a marginally stable nucleus confined within the cell.

We note that both in the experiments and the stochastic model of the WT, the cell-to-cell variation in robustness ($\sigma_{x}$) is much larger than in reliability (δ), suggesting that δ is under stiff control by the cell, while $\sigma_{x}$ is under a sloppier control.^82^^,^^83^^,^^88^ This, while expected, deserves further attention. The control of $\sigma_{x}$ is affected by the force-dependent catastrophe of MTs that we have included in our stochastic model—this acts as an adaptive feedback control, and possibly operates over a certain range of cell lengths. On the other hand, cellular control of δ is achieved by ensuring left (*L*) - right (*r*) symmetry in the MT-polymerizing forces. At present, we do not have a complete understanding of the molecular basis of this force correlation, which will likely involve anti-parallel crosslinkers such as Ase1^7^ and motor proteins such as Kinesin-14 or Dynein,^7^^,^^89^^,^^90^ an investigation of this cellular control mechanism is a task for the future.

### Limitations of the study

Our research findings and theoretical model suggest that achieving a high level of fidelity through the combination of two objectives—reliability and robustness—leads to minimal errors in septum positioning. However, it is important to note that the strict mathematical sense of optimality cannot be proven through experiments that involve perturbations in only a limited number of directions. Therefore, our demonstration of the optimality of the WT method is valid only within the scope of the perturbations studied.

## STAR★Methods

### Key resources table

**Table:** 

REAGENT or RESOURCE	SOURCE	IDENTIFIER
**Chemicals, peptides, and recombinant proteins**		

MBC (Carbendazim)	Sigma-Aldrich	378674

**Critical commercial assays**		

Gibson Assembly® Cloning Kit	NEB	E5510S

**Experimental models: Organisms/strains**		

h- Cut11:GFP:Ura Sid4mC:NatMx6	This Study	TP5070
h- ENVY-atb2:KanMx6	This Study	TP4070
h+ Sid4mC:NatMx6	This Study	TP4623
h- Sid4mC:NatMx6 Eatb2:KanMx6	This Study	TP4628
h- Eatb2:HphMx6	This Study	TP4878
h- Eatb2:HphMx6 Sid4mC:NatMx6	This Study	TP4880
h- Eabt2:KanMx6 Sid4mC:NatMx6 Cdc25-22	This Study	TP4756
h- Eatb2:KanMx6 Sid4mC:NatMx6 Wee1-50	This Study	TP4855
h- Eabt2:KanMx6 Sid4mC:NatMx6 Rsp1D:HphMx6	This Study	TP4763
h+ Eatb2:HphMx6 Sid4mC:NatMx6 Mto2D:KanMx6	This Study	TP4889
h- Eabt2:KanMx6 Sid4mC:NatMx6 Rsp1-1	This Study	TP4758
h+ Sid4mC:NatMx6 Eatb2:KanMx6 Ase1D:HphMx6	This Study	TP4629
h? Sid4mC:NatMx6 Eatb2:KanMx6 Klp5D:Ura Klp6D:His	This Study	TP4640
h- Eatb2:HphMx6 Sid4mC:NatMx6 Mcp1D:KanMx6	This Study	TP5056
h+ Sid4mC:NatMx6 Eatb2:KanMx6 Mal3D:HphMx6	This Study	TP4635
h- Eatb2:HphMx6 Sid4mC:NatMx6 Tip1D:KanMx6	This Study	TP5058
h? KanMx-pnmt1-GFP-Mal3 Sid4mC:NatMx6 Cdc25-22	This Study	TP5066
h+ Cut11:GFP:Ura Sid4mC:NatMx6 Rsp1D:HphMx6	This Study	TP5071
H? Cut11:GFP:Ura Sid4mC:NatMx6 Mto2D:KanMx6	This Study	TP5074

**Oligonucleotides**		

ATTCATCGATGATATCAGATCCACtagt	This Study	pfa6a-fwd
TTAATTAACCCGGGGATCCGTC	This Study	pfa6a-rev
gatccccgggttaattaaAGCATTTCCAGTCATATTTAACTC	This Study	patb2-fwd
ctttagacatTTCGAACGTCTTTCCAGGGT	This Study	patb2-rev
gttcgaAATGTCTAAAGGCGAGGAATTGTT	This Study	envy-fwd
atggatccTTTGTACAATTCGTCCATTC	This Study	envy-rev
attgtacaaaGGATCCATGAGAGAGATCA TTTCCATTCACGTTGGC	This Study	atb2-fwd
tccatgtcGCTGGCCGGGTGACCCGG	This Study	atb2-rev
cggccagcGACATGGAGGCCCAGAATAC	This Study	ter-fwd
ctgatatcatcgatgaatAACCGACAACTCAG TAGTCTTATT	This Study	ter-rev

**Software and algorithms**		

ImageJ	Schindelin et al.^91^	https://imagej.nih.gov/ij/
MATLAB	Version R2016a, The MathWorks Inc.^92^	https://www.mathworks.com/products/matlab.html

Simulation code	This manuscript	https://github.com/IJ-NCBS/MT_Sim_Code

### Resource availability

#### Lead contact

Requests for reagents should be directed to and will be fulfilled by the lead contact, Phong T. Tran (phong.tran@curie.fr).

#### Materials availability

Strains used in this study are available upon request.

### Experimental model and subject details

#### Genetics, cell culture, and strains

Standard yeast genetics methods are followed to insert fluorescent markers and to create strains.^93^ The EnvyGFP plasmid was a gift from the Bi Lab at the University of Pennsylvania. We use Gibson assembly to construct a pFa6a based plasmid to endogenously tag Atb2 with EnvyGFP inserted at the N-terminal and transformed it to the fission yeast cell. The list of primers used in this construct is given in the key resources table. A list of the strains used in the work can be found in the key resources table. In general, cells were maintained at 25°C on agar plates containing YE5S media. For microscopy based experiments, a small number of cells were inoculated in liquid YE5S medium a night before the experiment and incubated at 25°C under shaking for 16-18 hours, and then harvested when they reached an optical density of ∼ 0*.*5 ± 0*.*2 a.u.

### Method details

#### Microscopy and image analysis

Imaging was performed using a spinning disk confocal microscope. Specifically, we used a Nikon Eclipse Ti2 inverted microscope, equipped with a Nikon CFI Plan Apochromat 100x/1*.*4 NA objective lens, a Nikon Perfect Focus System (PFS), a Mad City Labs integrated Nano-View XYZ micro- and nano-positioner, a Yokogawa Spinning Disk CSU-X1 unit, and a Photometrics Evolve EM-CCD camera controlled by Molecular Devices MetaMorph 8.0 software. For GFP and mCherry imaging, we used solid-state lasers of 488,nm (100,mW) and 561,nm (100,mW).

To ensure experimental consistency, we used the CherryTemp microfluidics-based thermostat to precisely control the temperature during imaging. We aimed to analyze 20-50 cells in each case. To mount the samples, we immobilized them directly on a ready-made microfluidics chip-based setup (CherryTemp, CherryBiotech) using YE5S in 2% agar. After setting the temperature using the CherryTemp system, we waited for approximately 30 minutes for the cells to acclimate to the new conditions before proceeding with imaging, with the exception of the microscopy of temperature-sensitive mutants *wee1-50* and *cdc25-22*, for which we waited approximately 2 hours before imaging. The microscopy was performed at 35°C. The specific conditions for each type of microscopy experiment are provided below.

• Live cell microscopy and segmentation of SPB: A z-stack comprising a total of 7 focal planes spaced 1 μm apart was acquired in the brightfield channel (Exposure time 20 ms), as well as in the GFP (Exposure time 100 ms, EM Gain 400) and mCherry (Exposure time 100 ms, EM Gain 400) channels. The stack was acquired over a period of approximately 1 hour with a time interval of 15 s. We used the stack of brightfield images to segment cells based on the shift in the intensity of the cell boundary.^94^ To track a cell over the time-course, we linked the cell at time point *t* + 1 to time point *t* through maximum overlap. Although this method for segmenting and tracking the cells was very robust, we manually corrected the segmented cells whenever an error was found. To segment the Sid4-mCherry signal, we first constructed an image with maximum projection in the mCherry channel of the segmented cell images, removed background via thresholding, and found a 3x3 pixel window with the highest integrated intensity.
• Live cell microscopy and segmentation of Nucleus: We followed a similar procedure as described above, with the exception that the stack was acquired over a period of approximately 1 hour with a time interval of 30 s. We used the same procedure to segment the cell and SPB as described earlier. To segment the Cut11-GFP signal, we first constructed an image with maximum projection in the GFP channel of the segmented cell images, then binarized the images by selecting the pixels with the top 5% intensities and selecting the largest object.
• Live cell microscopy and segmentation of MT in EnvyGFP-Atb2 strains: A z-stack comprising a total of 13 focal planes spaced 0*.*5 μm apart was acquired in the GFP (Exposure time of 100 ms, EM Gain 400) and mCherry (Exposure time of 100,ms, EM Gain 400) channels. The stack was acquired for approximately 10 minutes with a time interval of 6 seconds. The cell was segmented by setting a threshold in the GFP channel images after background subtraction and taking a maximum projection. Following this, we constructed a color-combined image stack using the GFP and mCherry channels. The MTs that emanate from the SPB were manually segmented using the ImageJ segmented line tool (see Figure S7) and analyzed using custom script written in MATLAB.
• The number of MT bundles, total MT mass, and orientation were measured using static z-stacks (using the microscopy procedure described above). The number of MT bundles was counted manually. The quantification of the total MT mass and orientation was done using the following steps. First, we segmented the MT cytoskeleton using the ”Trainable Weka Segmentation” plugin in ImageJ.^95^ A manually curated dataset was used to train the classifier to distinguish between the MT cytoskeleton and cytosol. The segmented probability histogram was bimodal, and we used the value corresponding to the minima to segment the MT cytoskeleton and create a segmented mask. The MT mass was estimated by directly integrating the total intensity in a sum projection under the segmented mask. The orientations were calculated using the OrientationJ plugin in ImageJ^96^ under the segmented mask.
• Live cell microscopy and segmentation of MT in Mal3 strains: A z-stack comprising a total of 13 focal planes spaced 0*.*5 μm apart was acquired in the GFP (Exposure time 100,ms, EM Gain 400) and mCherry (Exposure time 100,ms, EM Gain 400) channels. The stack was acquired over a period of approximately 10 minutes with a time interval of 6 s. For each cell, we constructed two types of kymographs (see Figure S8): 1) a cell-wide kymograph was used to score catastrophe events associated with MTs that grow longer than 5 *μm* in length, and 2) a kymograph using a 2x50 pixel window centered around the SPB was used to score events that occurred on a short time scale (length *<* 5 μm). In both cases, we manually traced the trajectory of the MT growth and analyzed using custom script written in MATLAB.
• Microscopy and segmentation of septum: We incubated the cells at 35°C for 3 hours and then imaged them to quantify the septum positioning. As known, the *cdc25-22* cell does not initiate the mitotic phase at the restrictive 35°C. Thus, to quantify the septum position in *cdc25-22* cells, we incubated the cells at 35°C for 3 hours and then quenched the temperature to 25°C. Most of the cells undergo mitosis immediately and form a septum after 40 − 50 mins. To segment the septum position, we manually segmented the cell end and septum using the point-tool in ImageJ.

#### Stochastic model for MT-dependent nucleus centering

Here we discuss a model for microtubule (MT) pushing-force dependent nucleus centering to help us understand the diversity of phenomena observed in our experiments. The framework takes into account the motion of the nucleus because of stochastic forces arising due to MT polymerization against the cell wall. The model also accounts for the non-linear growth dynamics of MT, number, and orientation of MT, etc., inside the cell. Since the imaging plane is an x-y projection, we will take the cell to be a 2-dimensional rectangle of length 2*L* and width 2*R* (see Figure 7B). The nucleus is modeled as a rigid circle, and MT nucleation sites are situated at MTOCs located at the periphery which nucleates MTs with swivel points.

As a consequence, the nucleus undergoes translational dynamics according to,

(Equation 5) $$\frac{dX}{d\;t}=\frac{1}{\zeta^{T}}\left\{\sum_{i=1}^{N_{r}}f_{i}^{r}\left(\psi_{i},\theta_{i},t\right)+\sum_{i=1}^{N_{l}}f_{i}^{l}\left(\psi_{i},\theta_{i},t\right)+f_{th}\right\}\text{,}$$

where $X=\left[x,y\right]$ is the position vector of the centroid of the nucleus, and $\zeta^{T}$ is the hydrodynamic translational drag tensor (see below). On the right hand side, $f_{i}^{r}\left(\psi_{i},\theta ,t\right)$ and $f_{i}^{l}\left(\psi_{i},\theta ,t\right)$ are the forces due to the $i^{th}$ microtubule emanating toward the right (r) or the left (l) directions respectively, located at an angle ψ with respect to the SPB ($\psi_{SPB}=0$) and with an orientation θ with respect to the long axis of the cell. $N_{r}$ and $N_{l}$ are the number of MT-nucleation sites on the nucleus aligned towards the right or left sides respectively at time *t*. $f_{th}$ are random forces of thermal origin, with statistics $\left\langle f_{th}\right\rangle =0$ and $\left\langle f_{th}\left(t\right)\cdot f_{th}\left(t^{'}\right)\right\rangle =6k_{b}T\zeta^{T}\delta \left(t-t^{'}\right)\text{.}$

The polymerizing MTs also induce rotational dynamics on the nucleus,

(Equation 6) $$\frac{d\omega }{d\;t}=\frac{1}{\zeta^{R}}\left\{\sum_{i=1}^{N_{r}}T_{i}^{r}\left(\psi_{i},\theta_{i},t\right)+\sum_{i=1}^{N_{l}}T_{i}^{l}\left(\psi_{i},\theta_{i},t\right)+T_{th}\right\}\text{,}$$

where ω is the angle between the longitudinal axis of the cell and vector the connecting center of the nucleus to SPB (denoted as r). $\zeta^{R}$ is the hydrodynamic rotational drag tensor (see below). Akin to the forces, $T_{i}=r_{i}\times f_{i}$ is the torque applied by $i^{th}$ MT on the nucleus where $r_{i}=r_{nuc}\hat{n}$, where $r_{nuc}$ is the radius of the nucleus and $\hat{n}$ is the unit vector in the direction of $i^{th}$ MTOC. $T_{th}$ are random torques of thermal origin, with statistics $\left\langle T_{th}\right\rangle =0$ and $\left\langle T_{th}\left(t\right)\cdot T_{th}\left(t^{'}\right)\right\rangle =6k_{b}T\zeta^{R}\delta \left(t-t^{'}\right)\text{.}$

Before we proceed, we need to specify the form of the hydrodynamic drag tensors $\zeta^{T},\zeta^{R}$ that enters into the dynamical equations above. We first argue that the dominant mechanism of momentum dissipation in fission yeast is via cytoplasmic viscosity and not, as in mammalian cells, the actin cortex viscosity.^22^ This is because the actin cortex in fission yeast cell is very sparse^97^ and unlike the mammalian cell there is no connected actomyosin mesh during interphase. Furthermore, the smallest gap between the nuclear membrane and the cortex is around 0*.*3*μ*m. The drag arising from the cytoplasmic viscosity is an effective drag that has contributions from both the nucleus and attached MTs. The cytoplasmic drag is likely enhanced due to the presence of intracellular structures such as the ER. Despite the complex nature of the cytoplasmic fluid, no spatial dependence has been observed in the dynamics of small lipid droplets in fission yeast cytoplasm,^98^ suggesting that the yeast cytoplasm behaves like an isotropic Stokesian fluid. Consistent with this, experiments on tagged particle dynamics show the usual diffusive behaviour *R*^2^ ∼ *t*^*α*^, with *α* distributed close to 1 (with a median = 0*.*8).^98^^,^^99^ We note that recent experiments^100^ suggest that MT dynamics is strongly influenced by cytoplasmic viscosity, reinforcing our claim that the effective drag must have contributions from both the nucleus and the MT.

Since the dissipation from the nucleus and the MTs (taken as dashpots) appear in series, their individual translational drag coefficients combine as,

(Equation 7) $$\frac{1}{\zeta^{T}}=\frac{1}{\zeta_{nuc}^{T}}+\frac{1}{\zeta_{MT}^{T}}$$

while the rotational drag predominantly has contributions from the rotating nucleus alone. The drag coefficients of the nucleus (considered as a rigid sphere of radius *r*) in the cytoplasm of viscosity *η* is isotropic,

(Equation 8) $$\zeta_{nuc}^{T}=6\pi \eta r$$

and

(Equation 9) $$\zeta_{nuc}^{R}=8\pi \eta r^{3}$$

The translational drag coefficients of the MTs is anisotropic, with its parallel and perpendicular components given by

(Equation 10) $$\zeta_{MT}^{T,∥}=\sum_{i}\frac{2\pi \eta l_{i}\left(t\right)\cos\left(\theta_{i}\right)}{\ln\left(l_{i}\left(t\right)\cos\left(\theta_{i}\right)/2r_{MT}\right)-0.2}$$

and

(Equation 11) $$\zeta_{MT}^{T,⊥}=\sum_{i}\frac{4\pi \eta l_{i}\left(t\right)\sin\left(\theta_{i}\right)}{\ln\left(l_{i}\left(t\right)\sin\left(\theta_{i}\right)/2r_{MT}\right)+0.84}$$

where $l_{i}\left(t\right)$ is the length of the longest MT emanating from the nucleus perimeter from angle $\psi_{i}$ and $r_{MT}$ radius of the MT.^24^ The expressions Equations 10 and 11 are valid in the infinite rod limit, i.e. when $\frac{r_{MT}}{\left\langle l\right\rangle }\ll 1\text{.}$

The above forms of the hydrodynamic drag are approximations to the real system. Both the finite geometry of the yeast cell that confines the cytoplasmic fluid and the permeability of the nucleus as it moves through the incompressible cytoplasmic fluid, affect the form of the drag. Denoting the permeability of the nucleus by k, the drag coefficient reduces by $\zeta_{nuc}=\frac{6\pi \eta r_{nuc}}{1+k/2r_{nuc}^{2}}$.^101^ On the other hand, the drag on the nucleus increases because of geometric confinement. Treating the yeast cell as a cylinder of radius *R*, with the assumption of axial symmetry, the drag coeffcient follows $\zeta_{nuc}^{T}=\frac{9\pi^{2\sqrt{2}}\eta r_{nuc}}{4{\left(\frac{R-r_{nuc}}{R}\right)}^{5/2}}$, evaluated to the lowest order in $\frac{R-r_{nuc}}{R}$.^102^ Finally, we have treated the nucleus as a rigid sphere. The deformability of the nucleus can also change the coefficient of drag.

We have experimentally estimated the translational diffusion of the nucleus (using MBC treated cells) and found the translational diffusion length scale to be of order 10^−2^*μm* which is significantly smaller than the variation of the nucleus position because of active forces (which are of order 10^0^*μm*). Consequently, we neglect the effect of thermal forces in Equations 5 and 6.

The microtubules apply forces only when they are in contact with the cell boundary and this is given by

(Equation 12) $$f_{i}^{r}\left(\psi_{i},\theta_{i},t\right)=\left\{ \begin{array}{cc} f_{p} & \text{if}\;f_{p}\leq f_{e}\\ f_{e}\text{,} & \text{otherwise} \end{array}\right.$$

Where $f_{p}$ is the pushing force applied by MTs because of polymerization when MTs are in contact with the cell cortex and $f_{e}$ is the critical buckling force. $f_{p}$ is given by:

(Equation 13) $$f_{p}^{l}=-f_{s}\left(1-\frac{V_{f}^{+}}{V_{o}^{+}}\right)\hat{n}\Theta \left(x\left(t\right)+r\;\cos\left(\omega +\psi \right)-l_{i}\left(t\right)\cos\left(\omega_{i}+\psi_{i}+\theta_{i}\right)-L\right)$$

assuming that the applied force prominently affects the ‘on’ rate (the probability of a tubulin subunit to get intercalate at the tip of microtubule) of tubulin subunits (see Peskin 1993,^103^ Dogterom 1997^31^). Here f_s_ is the stall force (i.e. force at which the MT growth halts), $V_{o}^{+}$ and $V_{f}^{+}$ are the mean growth rate of MTs during the free-growth phase and during the contact-phase with cell boundary (while MTs applying pushing force), $l_{i}\left(t\right)$ is the length of the MT. $\hat{n}=\left[\cos\left(\theta_{i}\right),\sin\left(\theta_{i}\right)\right]$ is the unit orientation vector of the MT. The Heaviside theta function $\Theta \left(x\left(t\right)+r\;\cos\left(\omega +\psi \right)-l_{i}\left(t\right)\cos\left(\omega_{i}+\psi_{i}+\theta_{i}\right)-L\right)$ enforces the condition that microtubules only apply force when they are in contact with the cell boundary. $f_{e}$ is the critical buckling force require to buckle MT with flexural rigidity κ and is given by:

(Equation 14) $$f_{e}=\max\left\{ \begin{array}{c} \kappa \pi^{2}/l_{i}{\left(t\right)}^{2}\\ \kappa \pi^{2}/l_{io}^{2} \end{array}\right.$$

where $l_{io}$ is the length of MT which can be constrained inside the geometry of the cell after buckling. The inclusion of $l_{io}$ emulate constraints imposed by cell geometry on the configurations of buckled MTs.

The growth dynamics of microtubule is stochastic where microtubules show dynamic instability. During the growth phase, MT polymerize with rate $V_{o}^{+}$ and during the shrinking phase, MT depolymerize with rate $V^{-}$. The microtubule will catastrophe (i.e. jump from the growing to shrinking phase) such that the catastrophe time $\tau_{cat}$ has a distribution given by $P\left(\tau_{cat}\right)$. We also model force dependent reduction of catastrophe time. Here, if there is no force applied on MT then the catastrophe time $\tau_{cat}$ has a distribution given by $P\left(\tau_{cat}\right)$. In the presence of force, the catastrophe time is given by $\tau_{cat}\left(V_{+}^{f}\right)=\tau_{o}+\frac{\tau_{cat}\left(V_{+}\right)-\tau_{o}}{V_{+}}V_{+}^{f}$, where $\tau_{o}$ is the mean catastrophe time at $V_{+}=0$.^32^^,^^77^ The value of *τ*_*o*_ is not known *in vivo*, but it is found to be ∼ 25 sec in the reconstitution experiments (see Janson 2003^32^).

We use the Monte-Carlo integration approach to get a time-resolved solution to the above model. The approach has 2-steps: 1. Monte Carlo step– which evolves the growth dynamics of MT. 2. Numerical integration step – which updates the position of the nucleus. In the Monte Carlo step, either MTs length changes depending on their growth state or MTs switch from growing to shrinking state or vice versa. The switch from growing to shrinking state follow the distribution $P_{e}=1-\exp\left(-\Delta t/\tau_{T}\right)$, where $\tau_{T}=\frac{1-\int_{0}^{\tau_{cat}=T}P\left(t^{\prime }\right)dt^{\prime }}{P\left(\tau_{cat}=T\right)}$ is the mean catastrophe time of MT with age *T*,^78^ and Δ*t* is the time-step. The switch from shrinking state to growing state occurs instantaneously if the $l_{i}\leq 0$. In the Numerical integration step, we update the position of the nucleus using the forward Euler method for explicit integration. We initiate the calculation by assigning a fixed number of MT-anchoring sites which are distributed on the nucleus randomly. The number of nucleation sites (i.e. number of bundles) is equal to $\left(\left(N_{r}+N_{l}\right)÷4\right)$ (i.e. quotient of the fraction). The MTs are then randomly assigned to these sites and the MT-anchoring site with the maximum number of MT is considered the SPB to defining $\psi_{SPB}$. MTs orientation *θ* follow the experimentally determined orientation statistics of MTs and each time MTs switch from the shrinkage to growing state a new *θ* is assigned to an MT. The *θ* of the longest MT originating from the each anchoring site is considered in evaluation of the drag forces. Throughout this study, we assumed $N_{r}=N_{l}$ (unless stated otherwise). We started each simulation with the nucleus located on the tip of the cell and performed a simulation for ≈ 7200s (a generation time of fission yeast cell), unless noted otherwise.

We utilize $P\left(\tau_{cat}\right)$ obtained from Mal3 strain (*cdc25-22* GFP-*mal3* *sid4*-mCherry) and anticipated that the difference in catastrophe times seen in experiments can be attributed entirely to the force dependent change in velocities. Thus the model has only two free parameters - *κ* and *f*_*s*_. We systematically vary these parameters to search for a set of conditions which matches the experimental observations (see Figure S10). We see that that for *κ* ≈ 1*.*25*pNμm*^2^ the value of $\sigma_{x}$ matches with experimental observations, attributed to the dominance of buckling and *f*_*s*_ only becomes a limiting parameters. For *f*_*s*_, ranging from 4−6 pN, the ⟨*τ*_*cat*_⟩ and dwell-times also matches with the experimental observations.

### Quantification and statistical analysis

#### Segmentation and quantification of microscopy data

The segmentation and analysis were done using semi-automated scripts written in ImageJ macros and MATLAB.^91^^,^^92^ The specifics for segmenting each type of microscopy experiment are described along with the microscopy condition above in the method details section of STAR Methods.

#### Determining MT-catastrophe time distribution using bayesian inference

Arriving at the correct statistics of the MT growth dynamics is crucial for a description of the MT-driven processes. However, estimating MT growth dynamics by observing MTs (EnvyGFP-Atb2) has limitations – we can only reliably follow the growth dynamics of the longest MT emanating from a MT-bundle. This limits the length (and time) window to observe the MT’s catastrophe events, and consequently, the measured catastrophe length or time distributions only represents a truncated subset of the entire distribution. Moreover, the lengths (and time) window for observing the MTs depend on the MT number, orientation and cell length, which may introduce systematic biases in the analysis, or may result in sparse statistics. Instead of using conventional approaches, such as maximum likelihood estimate (MLE), an alternative method is to ask how well the data explains a set of model parameters. This is achieved by the Bayesian inference method.^48^ Bayes rule assigns the posterior probability on a set of distribution parameters (θ) given the observations (O) by:

(Equation 15) $$p(\theta |O)=\frac{p\left(O|\theta \right)\;p\left(\theta \right)}{p\left(O\right)}\text{,}$$

where p(θ) is the prior distribution of the model parameters, p(O|θ) is the likelihood function of the observations O given the parameters θ and $p\left(O\right)=\int p\left(O|\theta \right)p\left(\theta \right)\;d\theta$ is the marginal likelihood distribution of the observations. Apart from the dataset of observations **O**, we also require the following to evaluate the above expression: 1. a probability distribution function (e.g. exponential, gamma, etc.) which is parametrized by the set θ and, 2. a distribution of prior probabilities of p(θ).

We use a gamma distribution, defined by a step parameter (N) and a time-scale parameter T, to describes the MT catastrophe time ($\tau_{cat}$)^78^^,^^79^^,^^104^:

$$P\left(\tau_{cat}|N,T\right)=\frac{\tau_{cat}^{N-1}\;\exp\left(-\tau_{cat}/T\right)}{T^{N}\Gamma \left(N\right)}\text{,}$$

where Γ(*N*) is the gamma function. At *N* = 1, the gamma distribution reduces to an exponential distribution, as has been considered previously.^50^^,^^77^^,^^105^ We find that the gamma distribution is much more general, and hence more suitable to represent MT-regulator-driven changes in the distribution of catastrophe time. To validate the choice of the distribution, we use the GFP:*mal3* *sid4*:mCherry *cdc25-22* (Mal3 strain) strain to measure the *τ*_*cat*_. By incubating the strain at the restrictive temperature (35°C), we can measure the MT +end dynamics in very long cells (see Figure S8). The MT seldom reaches the tip of these cell, thus observation of MT dynamics is free from the effect of force. In our hand, the growth rate of MT is very similar to the growth rate observed in the EnvyGFP-Atb2 strains (WT). The cumulative distribution of catastrophe time is plotted in Figure 7C yields the best fit by a gamma distribution with *N* = 3*.*6 and *T* = 35*.*0s using Maximum likelihood estimate, in agreement with the reasoning presented above.

To construct the prior probability distribution p(N,T) on the model parameters (i.e., *N* and *T*), for the second part of the calculation, we use the following information. We assume that the catastrophe time distribution is established robustly by the cell, as we observe that the MT growth dynamics parameters are very similar across the many perturbations (see Table S1). Thus we use a prior biased by the parameters of the catastrophe time distribution seen in the Mal3 strain. To accomplish this, we combined the likelihood distribution for catastrophe time data from the Mal3 strain and a uniform distribution (a flat prior). The prior takes the form $p\left(N,T\right)∼W_{1}p\left(N,T|\;O\right)+W_{2}U,$ where *p*(*N,T*|*O*) is the likelihood distribution evaluated from Mal3 strain data (*O*) (see Figure S8), *U* is a uniform distribution and *W*_1_ and *W*_2_ are weights. The weights are selected to ensure that the ratio of maximum and minimum probabilities in *p*(*N,T*) is ≈ 10 (see Figure S8). However, a small deviation from this ratio does not lead to different results. We evaluated the posterior distribution $p\left(N,T|O\right)$ using the grid method on the support N∈[1:10],T∈[10s:500s]. The evaluated $p\left(N,T|O\right)$ for all the strains are given in Figure S9. Even with this bias in the prior distribution, the data from *mcp1*Δ, *mal3*Δ and, *tip1*Δ show the highest value of *p*(*N,T*|**O**) at *N* ∼ 1 (i.e. an exponential distribution), assuring that the method indeed allow deviations in the predicted parameters. In Figure 7, we also show the full distribution made using the value of *N* and *T* at their respective *max*(*p*(*N,T*|**O**)). Most interestingly, a truncated distribution made using the predicted parameters and with the experimentally observed support (i.e. *τ*_*cat*_ ∈ [*a,b*], where *a* and *b* respectively are minima and maxima of the observed *τ*_*cat*_) shows an excellent fit with the experimentally observed distribution.

#### Statistical analysis

Statistical analyses were performed as summarized in each figure. Wilcoxon rank-sum test was performed using MATLAB (Statistics and Machine Learning Toolbox) to determine the significance as indicated in figure legends.^92^ Results of statistical tests are indicated as follow: ∗∗∗∗ for *p* ≤ 10^−5^, ∗∗∗ for *p* ≤ 10^−4^, ∗∗ for *p* ≤ 0*.*005, ∗ for *p <* 0*.*05, ns is not significant.

## Data Availability

• Microscopy data reported in this paper will be shared by the lead contact upon request. • All original code has been deposited at Github and is publicly available as of the date of publication. The link is listed in the key resources table. • Any additional information required to reanalyze the data reported in this paper is available from the lead contact upon request.
