# Supplementary material for: Reliable and robust control of nucleus centering is contingent on nonequilibrium force patterns
Source: iScience. 2023 Apr 13;26(5):106665. doi: 10.1016/j.isci.2023.106665 (PMC10173738; doi:10.1016/j.isci.2023.106665)
Supplement: Document S1. Figures S1–S10 and Tables S1 and S2 [file mmc1.pdf]

iScience, Volume *26*

Supplemental information

Reliable and robust control of nucleus centering is contingent on nonequilibrium force patterns

Ishutesh Jain, Madan Rao, and Phong T. Tran

iScience, Volume ■ ■

Supplemental information

Reliable and robust control of nucleus centering is contingent on nonequilibrium force patterns

Ishutesh Jain, Madan Rao, and Phong T. Tran

1 Supplementary Tables

Table S1: **MT-growth dynamics parameters**, related to Figure 2-5 and 7. p-values are calculated with respect to WT using Wilcoxon rank sum test. $***$ for $p \leq 10^{-4}$, $**$ for $p \leq 0.005$, $*$ for $p < 0.05$, ns is not significant.

Strain	V_o^+ $(\mu m/min)$	V_o^- $(\mu m/min)$	V_f^+ $(\mu m/min)$	$\langle\tau_{cat}\rangle\pm$ S.D. (sec.)	$\langle\tau_{dwell}\rangle\pm$ S.D. (sec.)	n
WT	4.8 ± 1.4	-13.8 ± 5.9	3.0 ± 0.9	115.5 ± 36.9	45.5 ± 36.0	22
cdc25-22	4.4 ± 1.3(ns)	-15.5 ± 4.2(ns)	2.9 ± 1.6(ns)	148.4 ± 62.2($*$)	17.3 ± 25.0($**$)	28
wee1-50	5.0 ± 1.9(ns)	-11.2 ± 4.4(ns)	2.9 ± 1.6(ns)	90.2 ± 33.1($*$)	54.7 ± 32.7(ns)	32
rsp1Δ	5.4 ± 1.5(ns)	-13.3 ± 4.9(ns)	3.1 ± 1.0(ns)	95.7 ± 38.5(ns)	40.8 ± 30.4(ns)	29
mto2Δ	6.9 ± 3.4($**$)	-10.9 ± 6.0(ns)	3.2 ± 1.6(ns)	126.5 ± 45.4(ns)	64.4 ± 40.3(ns)	27
rsp1-1	4.4 ± 2.4(ns)	-16.7 ± 6.2(ns)	2.8 ± 0.8(ns)	154.8 ± 46.4($*$)	63.2 ± 39.4(ns)	19
ase1Δ	4.3 ± 1.2(ns)	-13.2 ± 5.8(ns)	2.5 ± 0.9(ns)	157.9 ± 41.1($**$)	51.8 ± 32.7(ns)	16
klp5Δ-*klp6*Δ	5.3 ± 1.7(ns)	-12.7 ± 4.4(ns)	3.0 ± 1.3(ns)	107.8 ± 31.8(ns)	45.1 ± 33.4(ns)	21
mcp1Δ	5.3 ± 3.2(ns)	-11.8 ± 5.2(ns)	3.9 ± 4.7(ns)	152.0 ± 101.8(ns)	64.3 ± 66.4(ns)	16
tip1Δ	4.2 ± 1.6(ns)	-12.7 ± 5.3(ns)	NA	88.2 ± 71.4($*$)	NA	10
mal3Δ	4.2 ± 0.9(ns)	-11.6 ± 7.3(ns)	NA	52.2 ± 23.6($***$)	NA	11

Table S2: **List of model parameters and their reference values**, related to Figure 7

Parameter	Definition	Reference value
$2L$	Length of the cell	Variable (for WT=$14\mu m$ see Fig. 2)
$2R$	Width of the cell	$3.2\mu m$
ψ_i	Angle of ith MTOC	Random uniform distribution
θ	Orientation of MT	Random with statistics of MT orientation in WT cells
r_{nuc}	Radius of the nucleus	$1.3\mu m$
η	Viscosity of the cytoplasm	$\approx 0.9pNs/\mu m^2$ [1–3]
r_{MT}	Radius of the MT	$0.013\mu m$ [a]
$N = N_r + N_l$	Number of MTs	18 (for WT cells[4])
f_s	Stall force for MT growth	Fitted
κ	Effective flexural rigidity of MTs	Fitted
V_o^+	Growth velocity of MTs	Table S1
V_o^-	Shrinkage velocity of MTs	Table S1
V_f^+	Growth velocity of MTs during dwell phase	Table S1
$P(\tau_{cat})$	Distribution of catastrophe times	See Fig. 7
ΔT	Time-step	1 sec.

[a] In our calculations, each bundle is made of upto 6 MTs, which may have a large radius toward the core of the bundle. However, small variation in the r_{MT} doesn't affect the results.

2 Supplementary figures

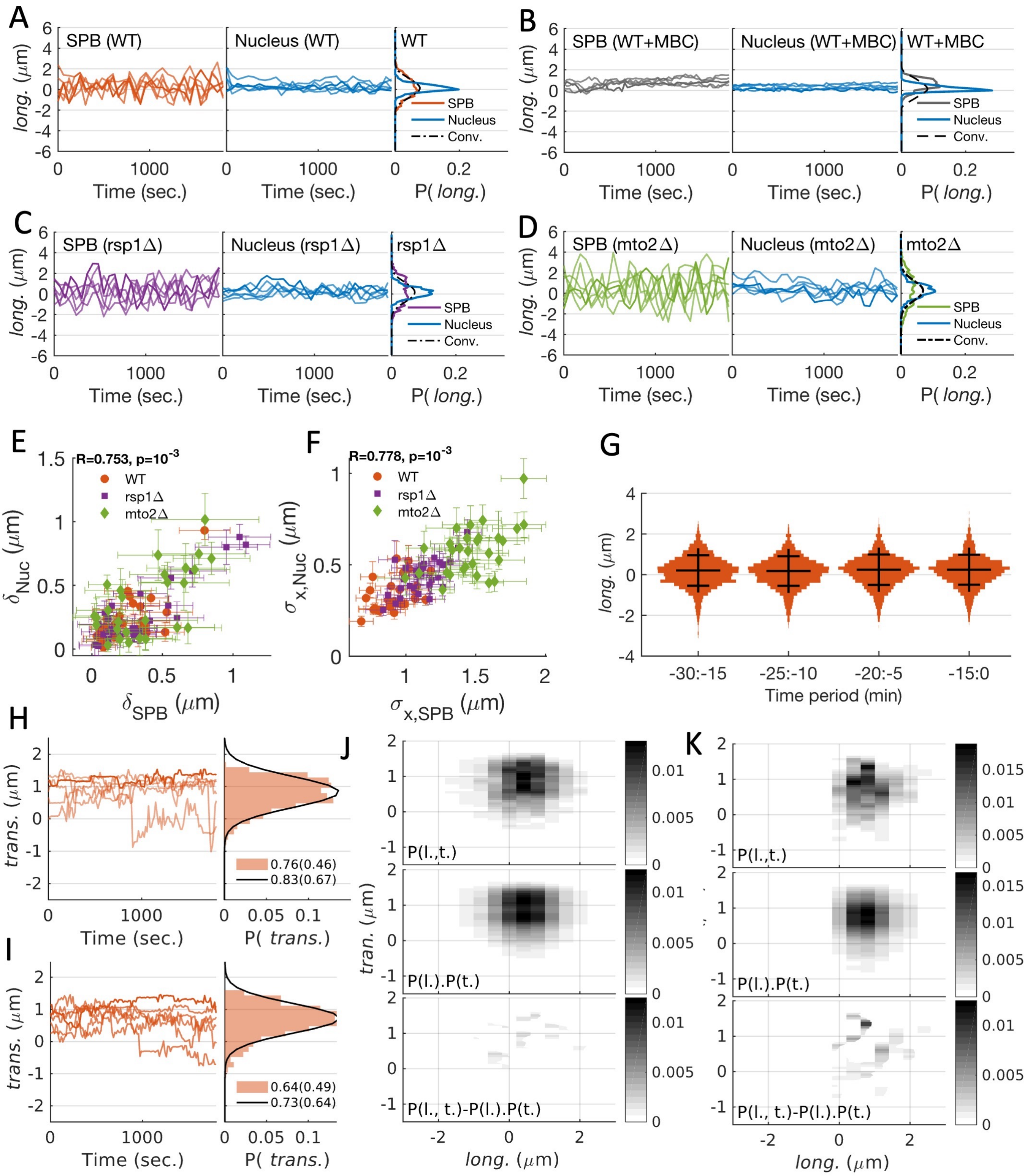

Figure S1: Nucleus centering in cells, related to Figure 1. **A.-D.** Examples of longitudinal displacement of SPB (left) and centroid of the nucleus (middle) w.r.t. the center of the cell in WT(A), MBC treated (WT+MBC)(B), *rsp1*Δ (C), and *mto2*Δ(D) cells. Nucleus fluctuations vanish upon MBC treatment (as seen previously, Tran2001 [5]). (right) Convolution of distribution of the nucleus centroid positions with a uniform distribution of points on the sphere surface fits well with the distribution of SPB positions in all strains. **E.-F.** Correlation between δ_{SPB} and δ_{Nuc} (E.) and $\sigma_{x,SPB}$ and $\sigma_{x,Nuc}$ (F). **G.** Time-series of SPB dynamics before the onset of mitosis is stationary. Violin plot of the distribution of SPB-positions at different time-periods (x-axis label) before mitosis. Error bars are standard deviation. **H.-I.** Examples of transverse displacement of SPB (left) and their distribution (right) in WT-cell (H) and with MBC treatment (I), respectively. **J.-K.** Joint distribution of the longitudinal and transverse position of SPB (top), the multiplication of marginal distribution of longitudinal and transverse position calculated independently (middle), and the subtraction of the two (bottom) for WT (J) and MBC treated (K) cells. These suggest that longitudinal and transverse positions are statistically independent, i.e., knowledge about the longitudinal position does not provide information about the transverse position and *vice versa*.

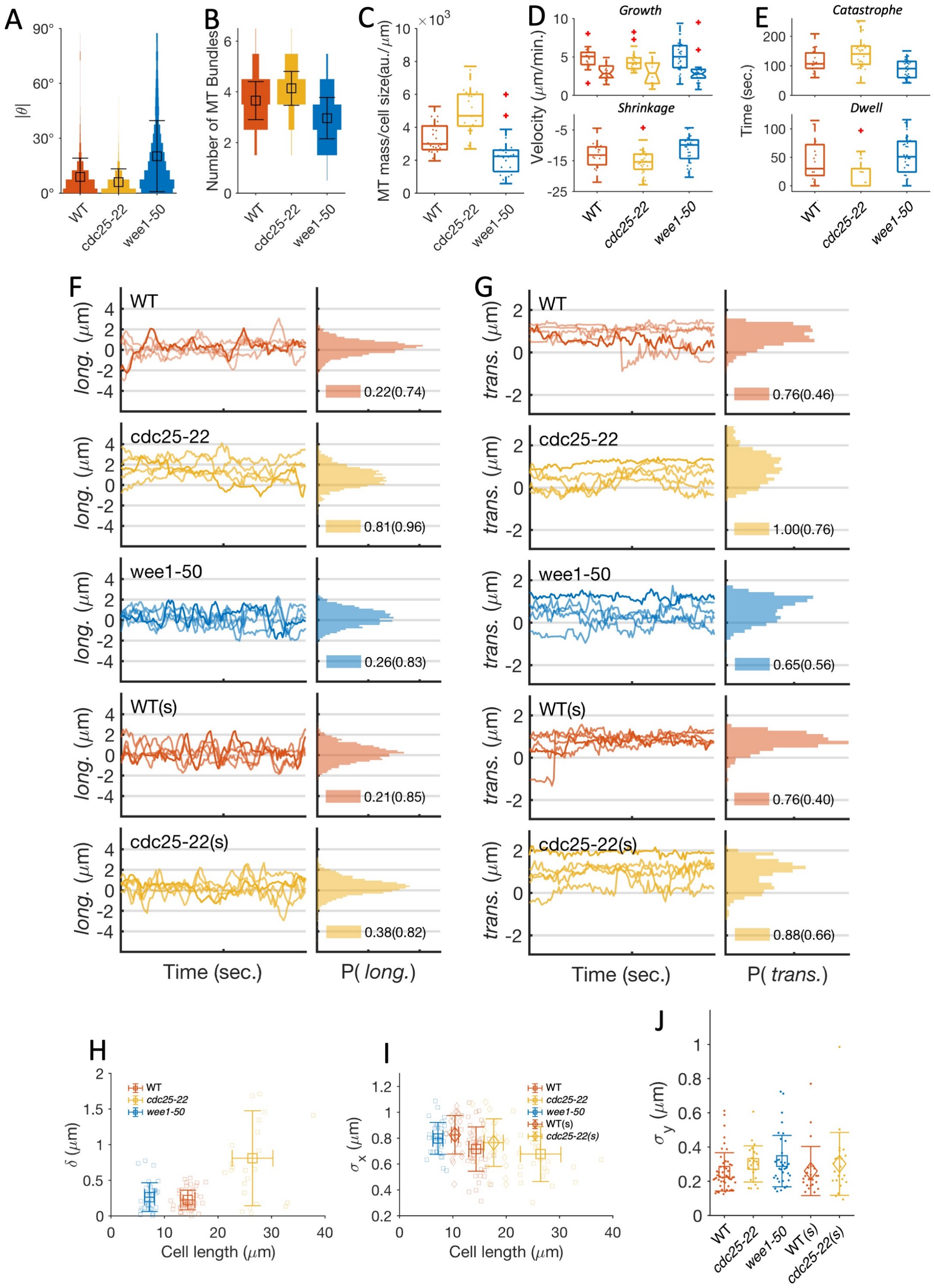

Figure S2: Properties of MT cytoskeleton and SPB dynamics in strains with cell-size perturbation, related to Figure 2. **A.-C.** Characterization of MT organization (also Fig. S6). **A.** The distribution of the local MT orientation θ, the local angle of MT filament relative to the longitudinal axis of the cell (error bars are SD). **B.** Distribution of the number of MT bundles with means equal to 3.7 (WT), 4.1 (*cdc25-22*), and 3 (*wee1-50*) (error bars are SD). **C.** MT-mass, measured from the intensity of MT fluorescence, scaled with cell length. **D.-E.** MT growth-dynamics parameters. **D.** Distributions of free growth velocity (top, boxed), growth velocities on contact (top, notched), and shrinkage velocity (bottom), are very similar across the cell strains (depicted by corresponding colors; see Table S1). **E.** The distribution of catastrophe times (top) and dwell times (bottom): show a dependence on cell length. **F.** Examples of time series of longitudinal SPB displacements relative to the cell center in different strains and conditions (left) and the histograms of longitudinal SPB positions in the population (right) (legend show means and (s.d.)). **G.** The representative trajectories of transverse fluctuations and histogram of transverse SPB positions (legend show means (s.d.)). Transverse fluctuation usually remains bound within a narrow range. Exceptionally we also see large, sudden movement. **H.-I.** Scaling properties of δ (H) and σ_x (I) with the cell length. **J.** Standard deviation in transverse displacement (σ_y) of SPB estimated for each cell. σ_y does not show any correlation with cell length.

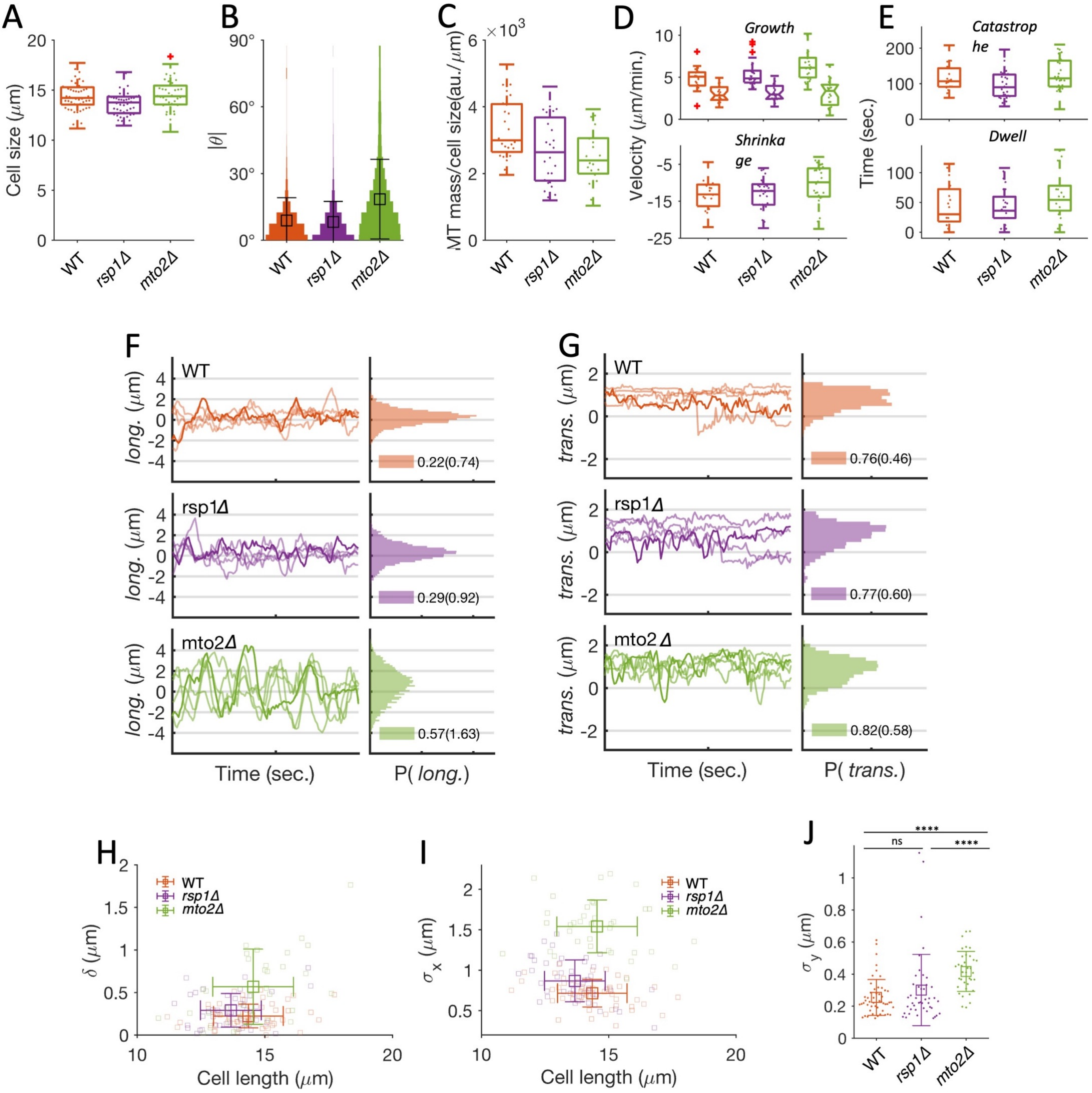

Figure S3: MT cytoskeleton and SPB dynamics in mutants affecting MT-bundles, related to Figure 3. **A.** Cell length distribution at the onset of mitosis (also Fig. S6). **B.** The distribution of the local MT orientation θ, the local angle of MT filament relative to the longitudinal axis of the cell (error bars are SD). **C.** MT-mass scaled with cell length, measured from the intensity of MT fluorescence. **D.-E.** MT growth-dynamics parameters. **D.** Distributions of MT growth velocity (top, boxed), MT growth velocities with contact (top, notched), and shrinkage velocity (bottom), for the different strains, are depicted by corresponding colors. (see Table S1 in SI). **E.** Distribution of catastrophe times (top) and dwell times (bottom). **F.** Examples of time series of longitudinal SPB displacements relative to the cell center in different strains (left) and the histograms of longitudinal SPB positions in the cell population (right) (legend show means and (s.d.)). **G.** The representative trajectories of transverse fluctuations and histogram of transverse SPB positions (legend show means (s.d.)). **H.-I.** δ (H) and σ_x(I) plotted against the cell length for WT, *rsp1*Δ, and *mto2*Δ strains. Changes in δ and σ_x with respect to the variation in MT-bundle numbers are independent of cell length. **J.** Standard deviation in transverse displacement (σ_y) of SPB estimated for each cell. σ_y is significantly large in *mto2*Δ cells.

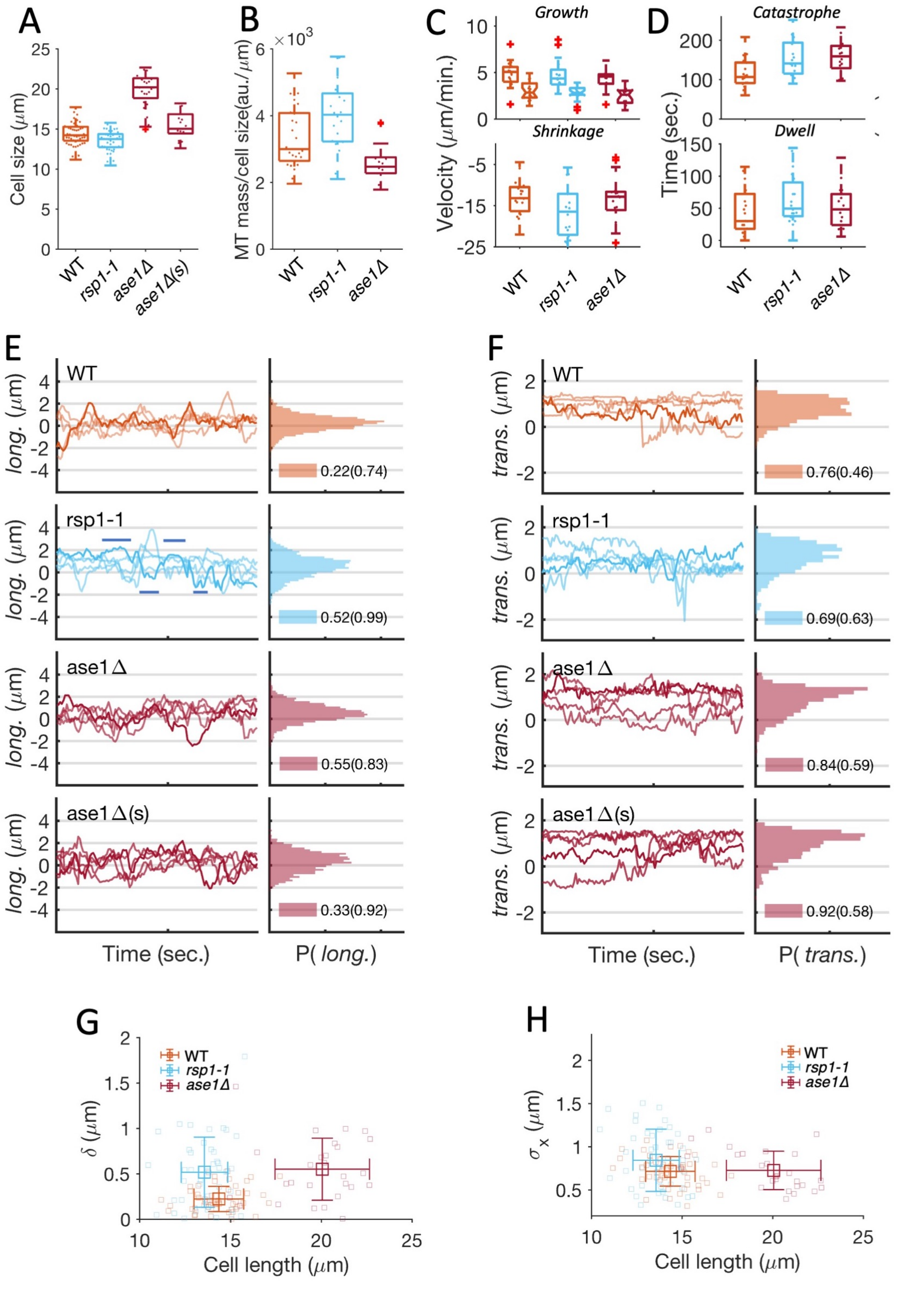

Figure S4: Properties of MT cytoskeleton and SPB dynamics in strains with altered orientational organization of MTs, related to Figure 4. **A.** Cell length distribution at the onset of mitosis (also Fig. S6). **B.** MT-mass, measured from the intensity of MT fluorescence scaled with cell length. **C.-D.** MT growth-dynamics parameters. **C.** Distribution of MT growth velocity (top, boxed), MT growth velocities with contact (top, notched), and shrinkage velocity (bottom), depicted by corresponding colors (see Table S1 in SI). **D.** Distribution of MT catastrophe time (top) and dwell time (bottom). **E.** Examples of time series of longitudinal SPB displacements relative to the cell center in different strains and conditions (left) and the histograms of longitudinal SPB positions in the cell population (right) (legend show means and (s.d.)). In *rsp1-1* mutants, many cells show pauses in SPB dynamics when the SPB is poleward localized (highlighted by horizontal lines). These states mostly reflect highly asymmetric, aster-like MT arrangement (see (E), *rsp1-1* panel). **F.** The representative trajectories of transverse fluctuations and histogram of transverse SPB positions (legend show means (s.d.)). **G.-H.** δ (G) and σ_x(H) plotted against the cell length for WT, *rsp1-1*, and *ase1*Δ strains.

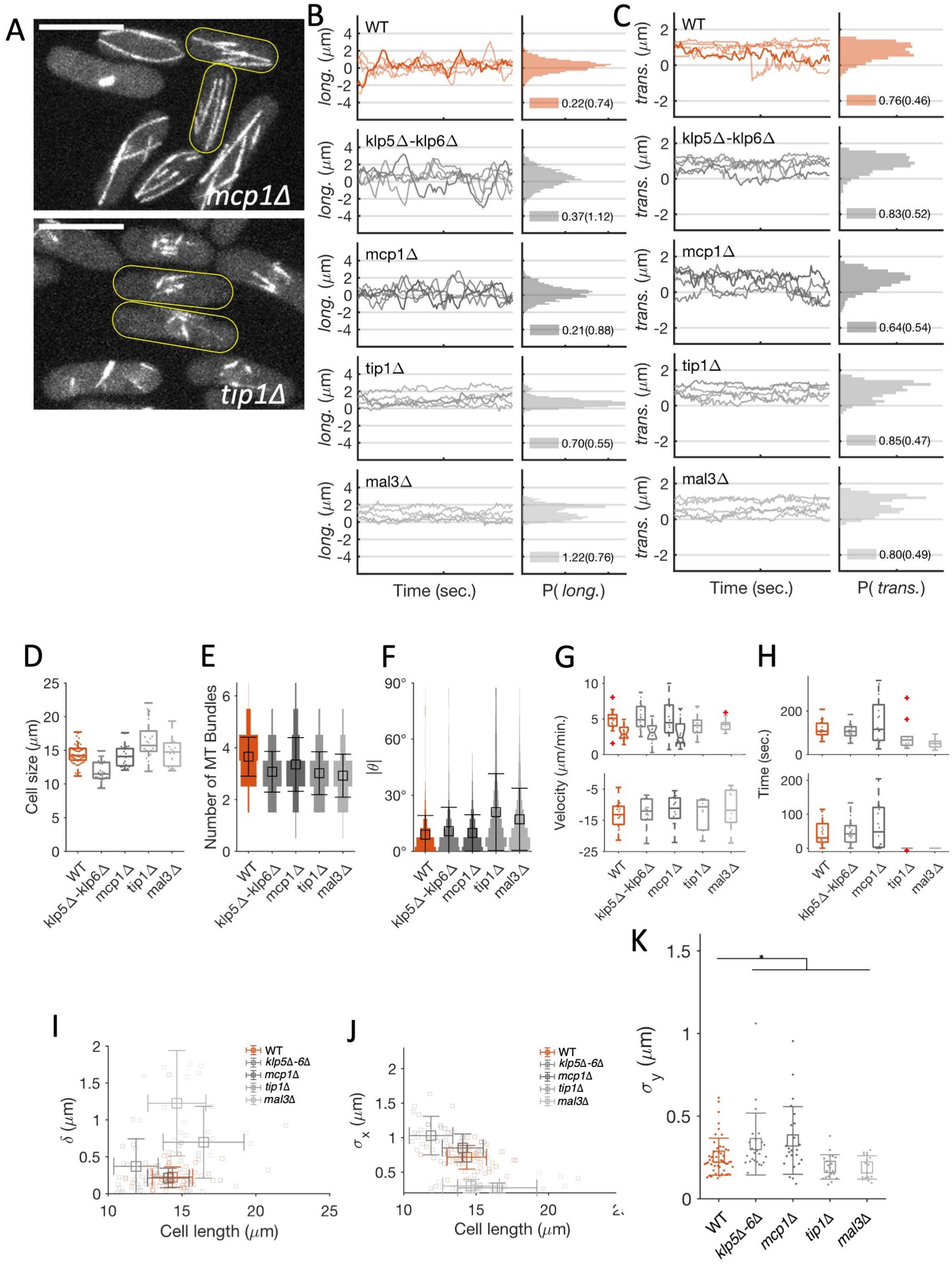

Figure S5: Characterization of MT dynamics mutants, related to Figure 5. **A.** Representative images showing MT organization in *mcp1*Δ (top) and *tip1*Δ cells (bottom). **B.** Examples of time series of longitudinal SPB displacements relative to the cell center in different strains (left) and the histograms of longitudinal SPB positions in the cell population (right) (legend show means and (s.d.)). **C.** The representative trajectories of transverse fluctuations and histogram of transverse SPB positions (legend show means (s.d.)). **D.** Cell length distribution at the onset of mitosis (also Fig. S6). **E.** Distribution of the number of MT bundles (error bars are SD). **F.** The distribution of the local MT orientation θ, the local angle of MT filament relative to the longitudinal axis of the cell (error bars are SD). **G.-H.** MT growth-dynamics parameters. **G.** Distribution of MT growth velocity (top, boxed), MT growth velocities with contact (top, notched), and shrinkage velocity (bottom), in the different strains, depicted by corresponding colors. **H.** Distribution of MT catastrophe time (top) and dwell time (bottom). **I.-J.** δ (I) and σ_x(J) plotted against the cell length for WT, *klp5*Δ-*klp6*Δ, *mcp1*Δ, *tip1*Δ, and *mal3*Δ strains. **K.** Standard deviation in transverse displacement (σ_y) of SPB estimated for each cell. σ_y is significantly large in *klp5*Δ-*klp6*Δ and *mcp1*Δ strains and significantly small in *tip1*Δ and *mal3*Δ strains.

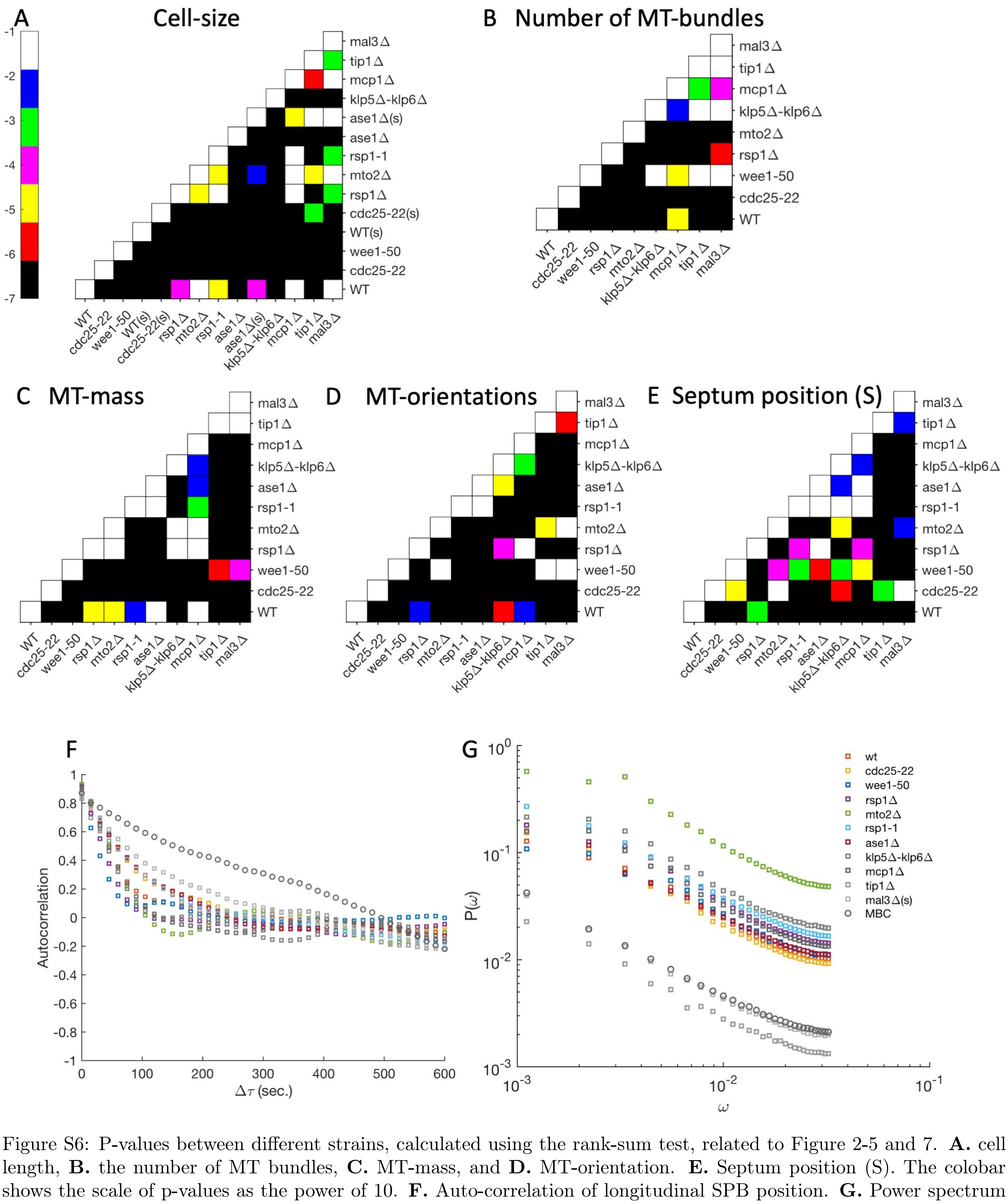

Figure S6: P-values between different strains, calculated using the rank-sum test, related to Figure 2-5 and 7. **A.** cell length, **B.** the number of MT bundles, **C.** MT-mass, and **D.** MT-orientation. **E.** Septum position (S). The colobar shows the scale of p-values as the power of 10. **F.** Auto-correlation of longitudinal SPB position. **G.** Power spectrum of longitudinal SPB position. The power spectrum does not peak at any particular frequency, suggesting that the SPB dynamics does not have a defined period.

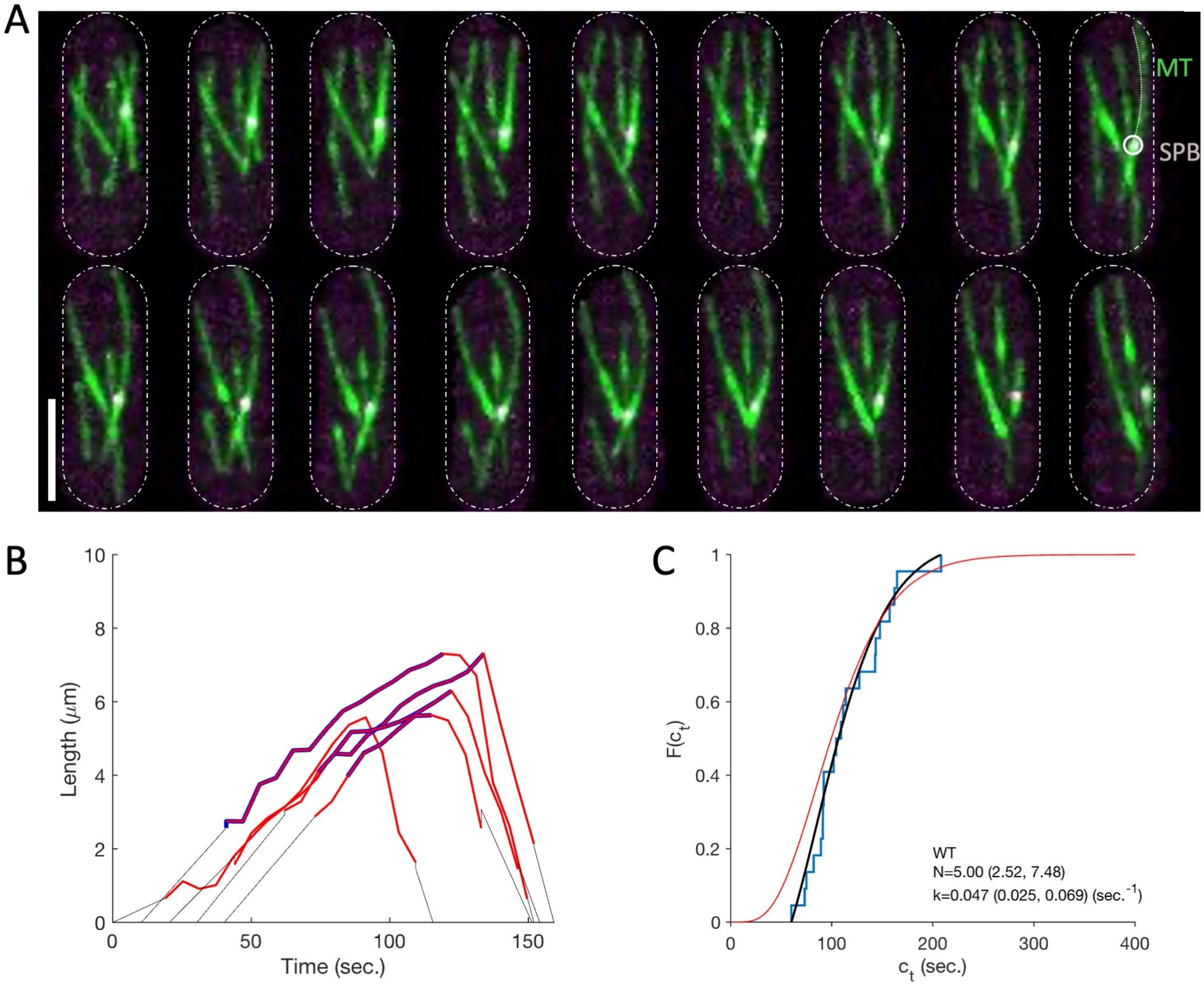

Figure S7: Measurement of MT growth dynamics parameters, related to Figure 7. **A.** A representative time-lapsed image sequence of a cell endogenously expressing EnvyGFP:Atb2 (MT, green) and Sid4:mCherry (SPB, cyan) in the WT background. Images are 6 seconds apart. While growing and making contact with the cell wall, MT show buckled morphologies and leads to large displacements in SPB. We traced the MT bundles length-wise to segment the growing MTs from a bundle originating from SPB. An example of such a trace is shown. **B.** Example of MT growth dynamics. Red traces show the measured length of MT from the SPB. The blue trace overlay represents the dwell phase. The dotted lines are an extrapolation of the growth trajectory evaluated using at least first (for growth) or last (for shrinkage) five data points for each MT. **C.** Empirical distribution function of catastrophe time observed in WT cells (blue curve). The black curve is fit using a maximum likelihood estimator using a truncated gamma distribution. The red curve shows the untruncated gamma distribution obtained using the fit parameters.

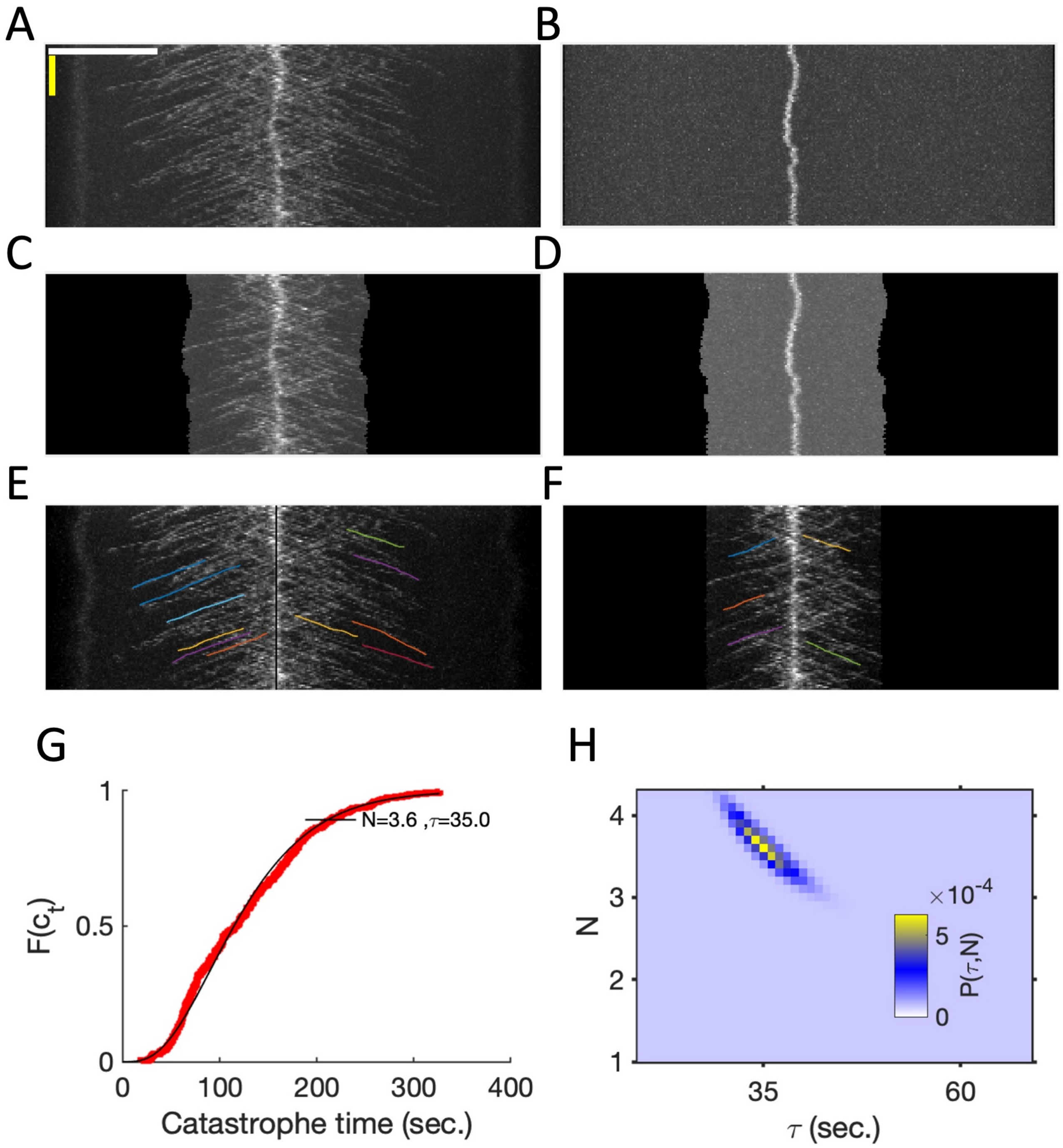

Figure S8: Catastrophe time distribution in long *cdc25-22* cells, related to Figure 7. **A.** Example of a whole-cell wide kymograph of Mal3 traces in a cell expressing GFP:Mal3 and Sid4-mCherry in *cdc25-22* background. We utilized the traces of comets with clearly visible ends to determine the catastrophe times (see *STAR Methods*). The white scale bar is 10 μm and the yellow scale bar is 120 sec. **B.** Sid4 (SPB) trace in the same cell. **C.-D.** Mal3(C) and Sid4(D) traces using a 2- by 50-pixel size rectangle around SPB. **E.-F.** Registered kymograph of Mal3 traces using SPB trace as reference. We manually segment only those Mal3 traces where the end is clearly visible. **G.** Red curve: Empirical distribution function of catastrophe times measure in long *cdc25-22* cells. The black curve shows the fitted gamma distribution using MLE. **H.** Informative prior used in Bayesian analysis of catastrophe times in various strains. The prior is constructed by combining a flat prior and the likelihood distribution derived from the Mal3 strain data set.

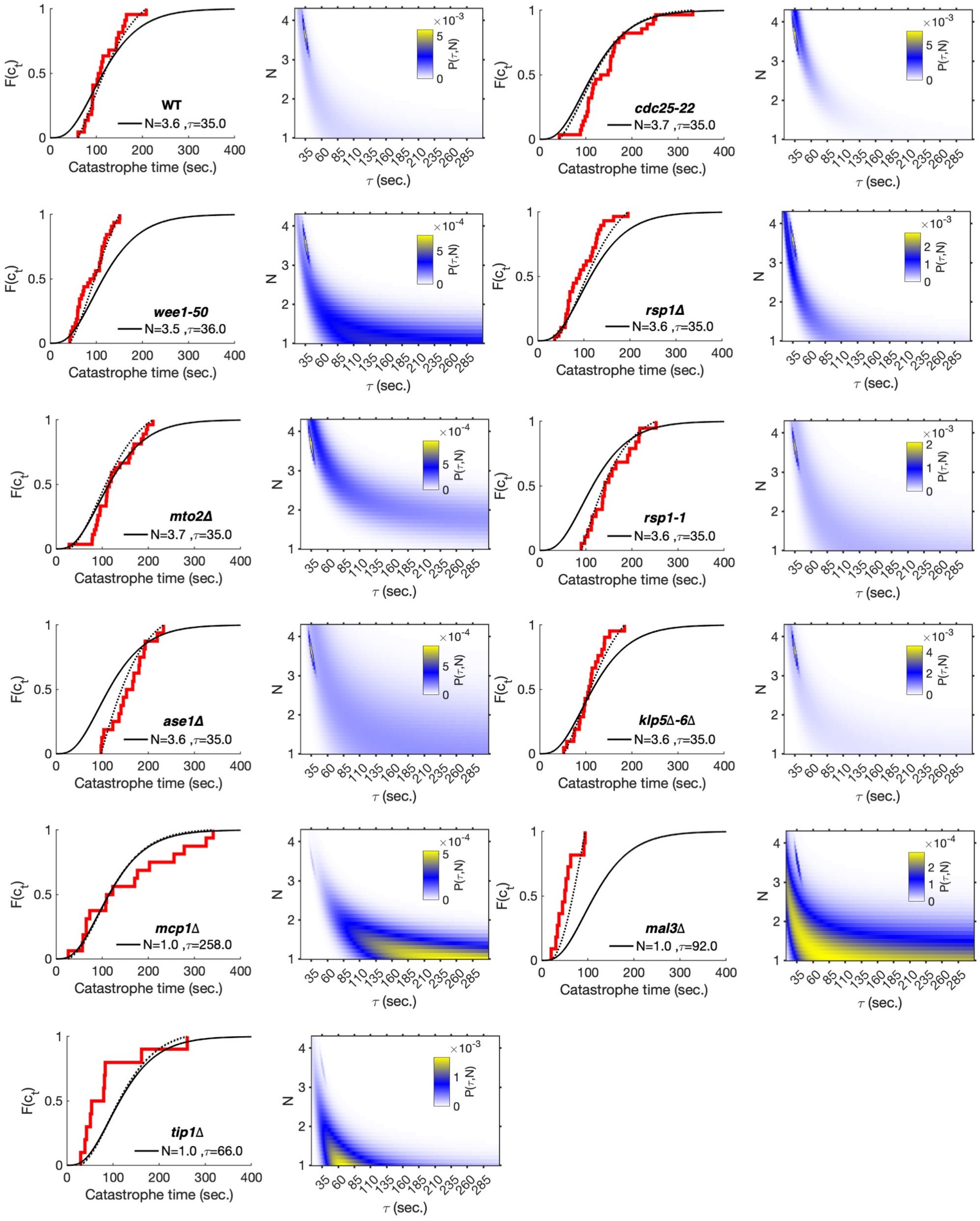

Figure S9: Related to Figure 7. For each denoted strain: (left) Parameters obtained using the Bayesian analysis of the catastrophe times. The red line shows the empirical probability distribution. The black curve shows the full distribution. The dotted curve is truncated distribution obtained using the estimated parameters (black curve) with truncation at the minimum and maximum of experimentally observed catastrophe times for respected strains. (right) Joint probability distribution of step parameter (N) and time-scale parameter (T) using catastrophe time data from the observation MT growth dynamics.

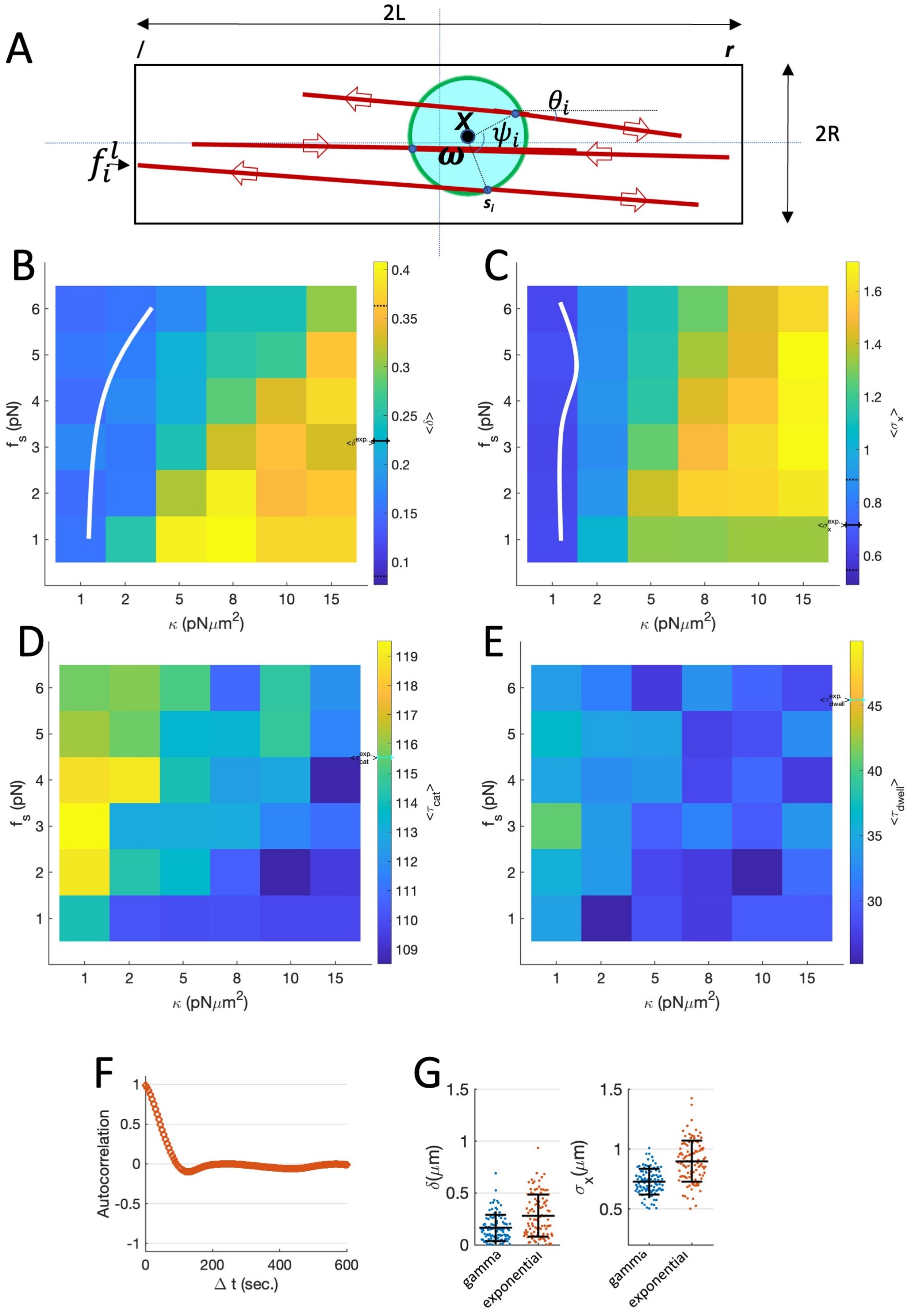

Figure S10: Stochastic model for nucleus positioning, related to Figure 7. **A.** Schematic depicting elements of the theoretical model for MT-driven nucleus centering. The MTs originate from MTOCs (blue dots) mounted on the periphery of a rigid nucleus (see detailed description in STAR Methods). **B-C.** Discrete contour plots for $\langle\delta\rangle$ (B) and $\langle\sigma_x\rangle$(C) were obtained by systematically varying the only two unknown parameters in the model (average of 100 independent instances). The solid and dotted lines on the color bar respectively mark the experimental mean and standard deviation. The white counter line in (B) and (C) corresponds to the experimental mean value of δ and σ_x respectively. **D.-E.** Discrete contour plots showing the mean catastrophe (D) and mean dwell time (E). The solid line on the color bar is the mean of experimental observation. **F.** Average auto-correlation from simulations using the parameter given in Fig. 7C. **G.** Effect of shape of the τ_{cat} distribution on reliable and robust nuclear centering. Statistics of δ (left) and σ_x (right) for the two catastrophe time distributions: gamma and exponential. The parameters in both cases are the same (as in Fig. 7C) except for the τ_{cat} distribution parameters. The exponential distribution has the mean τ_{cat} same as the gamma distribution.

SI References

[s1] Molines, A.T., Lemiere, J., Gazzola, M., Steinmark, I.E., Edrington, C.H., Hsu, C.T., Real-Calderon, P., Suhling, K., Goshima, G., Holt, L.J., et al. (2022). Physical properties of the cytoplasm modulate the rates of microtubule polymerization and depolymerization. Developmental Cell 57(4), 466–479.e6, doi:10.1016/j.devcel.2022.02.001.

[s2] Tolić-Nørrelykke, I.M., Munteanu, E.L., Thon, G., Oddershede, L., and Berg-Sørensen, K. (2004). Anomalous diffusion in living yeast cells. Physical Review Letters 93(7), 1–4, doi:10.1103/PhysRevLett.93.078102.

[s3] Foethke, D., Makushok, T., Brunner, D., and Nédélec, F.J. (2009). Force- and length-dependent catastrophe activities explain interphase microtubule organization in fission yeast. Mol. Sys. Biol. 5(241), 1–6, doi: 10.1038/msb.2008.76.

[s4] Höög, J.L., Schwartz, C., Noon, A.T., O'Toole, E.T., Mastronarde, D.N., McIntosh, J.R., and Antony, C. (2007). Organization of Interphase Microtubules in Fission Yeast Analyzed by Electron Tomography. Developmental Cell 12(3), 349–361, doi:10.1016/j.devcel.2007.01.020.

[s5] Tran, P.T., Marsh, L., Doye, V., Inoué, S., and Chang, F. (2001). A mechanism for nuclear positioning in fission yeast based on microtubule pushing. Journal of Cell Biology 153(2), 397–411, doi:10.1083/jcb.153.2.397.
